# Novel open reading frames in human accelerated regions and transposable elements reveal new leads to understand schizophrenia and bipolar disorder

**DOI:** 10.1038/s41380-021-01405-6

**Published:** 2021-12-23

**Authors:** Chaitanya Erady, Krishna Amin, Temiloluwa O. A. E. Onilogbo, Jakub Tomasik, Rebekah Jukes-Jones, Yagnesh Umrania, Sabine Bahn, Sudhakaran Prabakaran

**Affiliations:** 1 Department of Genetics, University of Cambridge, Cambridge, CB2 3EH UK; 2 Department of Chemical Engineering and Biotechnology, University of Cambridge, Cambridge, UK; 3 Leicester Cancer Research Centre, RKCSB, University of Leicester, University Road, Leicester, LE1 7RH UK; 4 Cambridge Centre for Proteomics, Department of Biochemistry, University of Cambridge, Tennis Court Road, Cambridge, CB2 1QR UK; 5 NonExomics, Inc, 2 Simon Willard Road, Acton, MA 01720 US

**Keywords:** Genetics, Neuroscience

## Abstract

Schizophrenia (SCZ) and bipolar disorder are debilitating neuropsychiatric disorders arising from a combination of environmental and genetic factors. Novel open reading frames (nORFs) are genomic loci that give rise to previously uncharacterized transcripts and protein products. In our previous work, we have shown that nORFs can be biologically regulated and that they may play a role in cancer and rare diseases. More importantly, we have shown that nORFs may emerge in accelerated regions of the genome giving rise to species-specific functions. We hypothesize that nORFs represent a potentially important group of biological factors that may contribute to SCZ and bipolar disorder pathophysiology. Human accelerated regions (HARs) are genomic features showing human-lineage-specific rapid evolution that may be involved in biological regulation and have additionally been found to associate with SCZ genes. Transposable elements (TEs) are another set of genomic features that have been shown to regulate gene expression. As with HARs, their relevance to SCZ has also been suggested. Here, nORFs are investigated in the context of HARs and TEs. This work shows that nORFs whose expression is disrupted in SCZ and bipolar disorder are in close proximity to HARs and TEs and that some of them are significantly associated with SCZ and bipolar disorder genomic hotspots. We also show that nORF encoded proteins can form structures and potentially constitute novel drug targets.

## Introduction

Although the heritability of both schizophrenia (SCZ) and bipolar disorder (BD) is ~70%—placing them among the most heritable mental health disorders [1–4], the corresponding polygenic risk scores explain only a fraction of genetic disease liability, for example, 7% in SCZ [5] relative to 64–81% heritability derived from family and twin studies. Moreover, putative individual genome-wide association studies (GWAS) risk alleles account only for a marginal increase in disease risk with odds ratios typically under 1.1 and differences in allele frequencies between cases and controls are often <2% [6, 7]. SCZ and BD; therefore, pose an evolutionary-genetic paradox because they exhibit strong negative fitness effects and high heritability, yet they persist at a prevalence of ~1% across all human cultures.

Past work has suggested that SCZ may be the result of human-specific brain evolution [8, 9] and some genes associated with the disease may have undergone positive selection [10, 11]. Moreover, studies have shown that mutations beneficial for human-specific cognitive prowess may also predispose an individual to SCZ [12, 13]. Similar hypotheses exist for BD, with one suggesting that BD is related to seasonal fluctuations in mood that may have increased reproductive fitness during the Pleistocene [14]. Hence, it is becoming increasingly clear that mutations beneficial to human cognitive abilities, including those related to disproportionately high consumption of energy by the human brain, might have not only been favored by natural selection but also increases the risk of SCZ [13]. In fact, the origin of both these disorders could be complex and likely governed by evolutionary mechanisms that are not mutually exclusive [15].

It is also possible that the genetic causes of these two disorders have not yet been identified because research to date has investigated primarily the conservatively defined regions of the genome called genes, more superficially, the genes that encode the known 20,000 proteins, which comprise just 1–2% of the human genome. However, recent evidence demonstrates that proteins can be encoded by genomic regions that cannot be defined as genes in the conservative sense. These unconventional and uncharacterized genomic regions are variously defined. Our own recent work has shown that RNA and proteins can be encoded pervasively throughout the genome, as observed in mouse neurons [16], cichlid fishes [17], malarial parasites [18], and different human cell types. More importantly, we have also shown that the proteins encoded by these unconventional and uncharacterized genomic regions, which we define as novel Open Reading Frames (nORFs), can form structures and be involved in different disease processes, including 150 rare diseases [19], and 22 cancers [20]. In addition, in Neville et al. [19], we show that many disease variants that have been often dismissed as “of uncertain significance” or “benign” would have to be re-classified based on our knowledge of these nORFs.

In addition to the above hypothesis that there might be more RNA and proteins than previously anticipated, in Puntambekar et al. [17], we showed that novel RNA and proteins emerge from accelerated regions of the genome of an organism. This observation may be relevant to SCZ and BD. We showed that nORFs emerge in accelerated regions of the cichlid’s genome that correlate with the time of their speciation, suggesting that nORFs might have enabled adaptive radiation in cichlids, resulting in almost 2000 species. As SCZ and BD are related to cognitive dysfunctions, cognition being a recently evolved ability, we set out to investigate whether nORFs that have been shown to have recently evolved or have been associated with human accelerated regions (HARs) could cast clues on the disease mechanism.

HARs are genomic segments that are highly conserved among non-human species but experience accelerated substitutions in the human genome [21]. Previous studies have demonstrated that HARs are mostly non-coding and likely to be regulatory elements responsible for human-specific traits [22]. For example, Pollard et al. [21, 23] identified 202 HARs through comparative genomics between human and non-human mammals and found that the most significant HAR (named HAR1A) is a novel long-non-coding RNA expressed in the development of the human neocortex [21, 23]. Other studies also have shown that genomic regions enriched with human-specific substitutions tend to be involved in the regulation of nervous system development and other developmental processes [24–28]. Capra et al. [29] assembled the HARs identified from different studies and predicted that at least 30% of the HARs were human developmental enhancers, and experimentally validated enhancer activity for 24 of 29 tested HARs. Many HARs are found in the introns of, and adjacent to, genes annotated with GO terms related to transcription and DNA binding [21–23, 26–29]. More HARs have been discovered in the years since their first description, often using different methods and by different groups [21, 23, 24, 30–33]. We curated a list of 4481 unique HARs split into three groups based on the extent of their conservation (Supplementary Table 1) and verified that they are present in all chromosomes (Supplementary Figure 1). vHARs are HARs conserved in vertebrates, mHARs are HARs conserved in mammals, and pHARs are HARs conserved in non-human primates. Of the 4481 unique HARs, 45.4% are vHARs, 11.0% are mHARs, and 43.6% are pHARs.

The human-centric nature of HARs led to investigations into their link with SCZ. pHARs were found to be enriched in SCZ-associated loci and pHAR-associated SCZ genes were found to be under stronger selection pressure than other SCZ genes [34]. In addition, mutations in HARs have been found to contribute to altered cognitive behavior [35], suggesting some importance in neural function. However, to our knowledge, HARs have not been systematically examined in any of the psychiatric diseases. The recent results from the PGC meta-analysis provide a novel opportunity to investigate systematically the role of HARs in SCZ.

Another group of genomic features that have been shown to regulate gene expression is transposable elements (TEs). TEs come in two classes. Class I are retrotransposons, consisting of long terminal repeats (LTRs), which include human endogenous retroviruses, and non-LTRs, which include long interspersed nuclear elements (LINEs), short interspersed nuclear elements (SINEs), and SINE/VNTR/Alu elements (SVAs). TEs have been shown to act as regulatory elements by a variety of means [36]. They can regulate nearby gene expression mainly by acting as alternative promoters, but they can also act as enhancers and other regulatory elements [37]. Their action as enhancers is particularly interesting as enhancers can arise through the insertion of TEs [38]; it is feasible that some HARs arose through TE insertion. TEs can be a source of non-coding RNAs and can act as insulators or boundary elements, splitting the genome into 100kb-1Mb domains of active and inactive transcription by preventing the spread of heterochromatin. Indeed, many TEs (especially SINEs) harbor binding sites for factors (CTCF, TFIIIC) that confer insulator activity and organize nuclear architecture. Furthermore, chromatin-based repression of TEs [39] impacts the expression of nearby loci [40]; when said repression fails, neighboring loci may be expressed together with the corresponding TEs. There has been growing evidence for the association between TEs and SCZ and BD including reports of increased transcription of human endogenous retroviruses (HERVs) in patients diagnosed with SCZ and BD [41–45]. Overexpression of HERV-W env may upregulate several SCZ-associated genes in U251 glioma cells [46] and downregulate *DISC1*, a gene known to be disrupted in SCZ, in humans neuroblastoma cells [47]. These findings suggest that human-specific evolutionary changes may have contributed to the genetic architecture underlying SCZ traits in modern human populations.

HARs and TEs are two classes of genomic regions that can play a role in the regulation of nearby genes by acting as enhancers, alternative promoters, or coding for short RNA sequences that influence the expression of nearby genes. HARs were implicated in SCZ development by Xu et al. [34] and several TE families have been shown to be either differentially expressed (DE), methylated, or otherwise regulated in SCZ and BD [42, 48, 49]. Although the association of HARs and TEs with SCZ- and BD-associated genes has been investigated to some extent, less attention has been placed on non-coding regions, especially nORFs and their transcriptional and translational end products. nORFs are present in both coding and non-coding regions of the genome and may be biologically regulated [16]. nORFs can encode functional protein-like structures that may play a role in disease [20]. For example, a novel transcript was found to be expressed in a 22q11 deletion syndrome brain [50].

In this study, we hypothesize that components of the genetic architecture of SCZ and BD are attributable to human-lineage-specific evolution and that some of these components may have not yet been discovered because of our conservative definition of a gene and because of analyzing genomic, transcriptomic, and proteomic data in silos. To investigate this, we performed a genome-wide evolutionary assessment of the overlap between nORFs present in HARs and in SCZ and BD-associated loci.

We systematically mined SCZ and BD data sets from the PsychECNODE consortium [51] to detect the expression of nORFs with previously determined evidence of translation, which we curated in our nORF database. Following that, we assessed the relationship and association between differentially expressed nORFs (DE nORFs) and HARs and TEs, and their enrichment in SCZ and BD-associated loci. We were particularly interested in identifying DE nORFs present in pHARs associated with SCZ and BD loci. We also investigated the correlation of HARs or TE transcript expression and nORF transcript expression to identify any potential regulation. In addition, for a smaller subset of samples, we were able to show evidence of translation of nORFs. Finally, and more importantly, as we did in Erady et al. [20] and Gunnarsson and Prabakaran [18], we predicted structures for some of the nORFs implicated in both the disorders to demonstrate that they may serve as novel drug targets. Thus, our work highlights interesting molecular mechanisms that have been previously missed and we anticipate that this will lead to novel treatments.

## Methods

### Creation of nORF data set

We used nORFs obtained from two sources—nORFs.org [19] and RPFdbv2.0 [52]; however, nORFs from RPFdbv2.0 were further processed. In brief, the expression of nORFs was compared with canonical ORFs (cORFs) from 53 studies (with 353 samples), downloaded from RPFdbv2.0 across 11 human cell lines [52]. The 353 samples were divided into 11 groups based on cell types. Actively translated ORFs with clear sub-codon phasing or triplet periodicity footprints were detected using the RibORF tool for each study [53]. Further, each ORF entry was appended with its corresponding annotations: genomic position, strand, ORF category (one of: canonical, truncated, extended, uORF, overlapping uORF, internal, external, polycistronic, readthrough, non-coding transcripts), length of encoded amino acid, ribosome profiling abundance (RPKMs, raw read counts) and the transcript to which the ORF maps (probable transcript from which ORF is translated). Raw read count abundance for each ORF was then converted to Transcript per million (TPM) values for downstream analysis.

Mean and standard deviation (SD) of Ribo-seq expression TPMs for all 353 samples in each of the 11 groups were compared between the canonical and the “non”-canonical ORFs. Mean values were divided into exactly 4000 quantiles with every quantile containing the same number of ORFs. Within each quantile, the SDs were compared between nORFs and cORFs of consequently similar means. ORFs with SDs less than the median SD of cORFs were termed low-noise ORFs as described in Erady et al. [20]. In all, 101,797 such low-noise nORF entries were added on to previously curated nORFdbV1, and further, duplicates were removed and classification was performed as described in Neville et al [19]. Any nORF classified as in-frame to the CDS of a cORF was removed except for when an annotation such as readthrough, extension, uORF, or truncation was determined using the RibORF tool, leading to a final of 248,135 nORF entries. Bedtools getfasta was used to extract the corresponding nucleotide sequence for the new nORF entries using GRCh38 DNA primary assembly (ftp://ftp.ensembl.org/pub/release-96/fasta/homo_sapiens/dna/) with parameters “name”, “s”, and “tab” specified. nORF sequences identified using RibORF were translated using Biostrings package in R, which was appended to the curated amino-acid sequences of nORFs. The results of this analysis are illustrated in Supplementary Figure 2.

### Description of the data sets

Two data sets were used in the study and they are as follows: 1. transcripts from the PsychENCODE data set and 2. transcripts and mass spectra from the Stanley Medical Research Institute (SMRI) consortium data set. The details of the data set and the analysis performed using them are given below. In brief, the PsychENCODE transcriptomic data were used to identify and quantify nORFs, HARs, and TEs, and the SMRI transcript data (shared by the SMRI consortium directly with us and published by Kim et al. [54]) and the proteomic data (kindly shared by Dr. Michael G. Gottschalk and Dr. Hendrik Wesseling and published by Gottschalk, MG et al. [55]) were analyzed using the proteogenomic workflow to demonstrate evidence of translation of nORFs in a subset of samples.

### Identification of nORF transcripts in PsychENCODE data set

We chose three out of the eight studies, namely BrainGVEX, CMC, and CMC_HBCC, which are part of the PsychENCODE consortium [51], for our analysis. These three studies were selected based on the availability of total RNA-seq data from SCZ, BD, and control (CNT) adult post-mortem brain samples (Supplementary Table 2). The total number of samples used in the analysis were 1340 patient samples – 731 CNT, 428 SCZ, and 188 BD. The processed BAM files and RNA-Seq by Expectation-Maximization (RSEM) count files are available under freeze 1 and freeze 2 of the PsychENCODE Consortium. Briefly, CNT, SCZ, and BD samples were isolated from the DLPFC, primarily BA9 and BA46, as part of eight different studies. For our analysis, RNA-Seq results of three studies: CMC_HBCC, CommonMind, and BrainGVEX, with samples from CNT, SCZ, and BD brain samples were used. RNA-Seq reads were aligned to the hg19 reference genome using STAR 2.4.2a. Gene- and isoform-level quantifications were performed using RSEM v1.2.29.

Correlation analysis of gene expression between samples showed a higher correlation between samples from the same study group than between samples from different study groups (Supplementary Figure 3A). Moreover, principal component analysis (PCA) of both gene and transcript expression for these samples was performed to identify batches/clusters corresponding to the different study groups (Supplementary Figure 3B). Clearly, this analysis revealed the considerable batch effects that were accounted for in downstream analyses.

To confirm that the gender metadata labeling for our samples was correct, we conducted two analyses. First, using the average chromosome Y (chrY) gene expression levels, samples were split and plotted according to their metadata information to confirm that those annotated females had 0 or low chrY gene expression. Second, hierarchical clustering of XIST gene expression using R functions hclust (method = “single”) and cutree, allowed us to compare the two resultant clusters with their respective gender metadata (Supplementary Figures 4 and 5).

### Identification of transcribed nORFs in PsychENCODE data set

GRCh37-based transcript and gene coordinates for 1340 neuropsychiatric samples from the BrainGVEX, CMC, and CMC_HBCC studies were obtained from the PsychENCODE consortium. Transcript expressions were filtered to retain those with TPM > 0.1 in at least 10% of the samples. In addition, transcripts from the Y-chromosome pseudoautosomal regions (PAR) were removed. GffCompare (v0.11.5) mapping was performed between the nORF and sample transcript coordinate as described in Erady et al. [20]. The results file was further filtered as specified in https://github.com/PrabakaranGroup/norfs_in_neuropsychiatric_disorders. Transcripts containing nORFs with biotype not equal to “protein-coding” were retained. We also performed this analysis with TPM cutoffs of 1, and 10.

### Identification of DE nORFs

To identify underlying covariates that could affect the DE analysis between SCZ, BD, and CNT, we used multivariate adaptive regression spline (MARS) [56] and surrogate variable analysis (SVA) [57] using the earth and sva package, respectively, in R. Sample transcript count values generated using (RSEM) were normalized using trimmed mean of M-values (TMM) method with edgeR. Earth model with linpreds set to true was run 1000 times and covariates identified at least half of the time were retained. seqPC1-3, seqPC5-7, seqPC10-14, seqPC16, seqPC18-25, seqPC27-29, RIN, RIN.squared, age, batch, and individualIDSouce were identified as covariates which were then accounted for during differential expression (DE) analysis.

DE analysis was performed through a linear mixed-effects model using nlme package in R, with the above set as fixed effects and individual id as a random effect [58]. EdgeR TMM normalized and log_2_(CPM (expression) +0.5) counts were analyzed for DE between CNT and BD and CNT and SCZ. Transcripts that were identified as DE at an FDR < 0.05 after Benjamini–Hochberg correction of the associated *p* values, were further evaluated for nORF presence using the GffCompare workflow mentioned previously.

We identified the number of DE nORFs obtained with different TPM cutoffs (Supplementary Table 3). We also investigated whether the number of DE nORFs obtained were more or less expected by chance using a two-tailed fisher’s exact test with an arbitrary *p* value threshold of 0.05. For SCZ the odds ratio was 1.266 at a *p* value of 0.09, and for BD the odds ratio was 1.407 at a *p* value of 0.04408.

### Potential functional inferences of nORFs from amino-acid sequence

For the 248,135 curated nORFs, Gene Ontology (GO) terms were obtained from equivalent InterPro [59] IDs generated using InterProScan5 run on the galaxy server [60]. Of the total input, 27,430 nORFs with a total of 62,700 corresponding GO terms were identified. Further analysis revealed that of the 3103 nORFs identified as transcribed in SCZ and BD samples, 49 nORFs had associated GO terms. Similarly, 2 out of 44 and 13 out of 61 DE nORFs in BD and SCZ, respectively, had corresponding GO terms. For the translated nORFs, 17 out of 21 had GO terms. GO term enrichment for each of these nORF categories was conducted using the GOEnrichment tool on the galaxy server. The required.OBO file for this run was obtained from http://www.obofoundry.org/ontology/go.html. Analysis was conducted at a *p* value cutoff of 0.01 with Benjamini–Hochberg multiple testing correction enabled.

### Enrichment analysis of DE nORFs within SCZ and BD loci

We evaluated the presence and enrichment of DE-transcribed nORFs within SCZ and BD-associated loci using an annotation and enrichment tool GLANET [61], which uses random sampling to calculate enrichment of genomic elements within the input query. In addition, we investigated enrichment of certain DNase I hypersensitive sites (DHS1), histone modifications, and transcription factors (TFs) within the transcribed and DE nORF cohort. SCZ-associated high confidence regions were obtained from PsychENCODE resource (http://resource.psychencode.org/). For BD, associated loci coordinates were taken from Stahl et al. [7]. SCZ CNVs were curated by Guyatt et al. [62].

### Identification of unique HARs

In all, 4481 unique HARs were compiled from seven papers discussed in the introduction [21, 23, 24, 30–33], which have identified HARs—the papers and the notation used to refer to each set of HARs henceforth, are outlined in Supplementary Table 1. The genomic coordinates of HARs were mapped to hg19/GRCh37 genome assembly where required, using the LiftOver tool (Supplementary Figure 1). The seven coordinate files for HARs and the merged list are available in norfs_in_neuropsychiatric_disorders/supplementary_data/ on GitHub.

### Association of nORFs with HARs

In all, 3103 nORFs were identified to be DE in the BrainGVEX, CMC, and CMC_HBCC neuropsychiatric samples. These nORFs are defined to be associated with a HAR if the HAR overlapped the nORF or regions extending 100 kb upstream or downstream of the nORF. This association distance is in accordance with previous work [34], although a previous study looked at association within 1 kb [30] and another study found that 52% of non-coding HARS examined in the study are located within 1MB of a developmental gene and 59% are within 1 Mb of a gene DE between humans and chimpanzees [29]. An nORF associated with a HAR is referred to as an nORF-HAR.

### Stratification of SCZ and BD-associated SNP loci

SCZ-associated SNPs [63] and BD-associated SNPs [7] were stratified by *p* value (*p* < 10^−2^; *P* < 10^−3^; *P* < 10^−4^; *P* < 10^−5^; *P* < 10^−6^; *P* < 10^−7^) (Supplementary Figure 6). To summarize linkage-disequilibrium (LD)-dependent associations between SNPs, these sets of SNPs were clumped in PLINK 1.9 [64] using LD-based clumping and data from 1000 Genome’s EUR population (The 1000 Genomes Project Consortium, 2015). Clumping produces LD-independent sets (“clumps”) of SNPs, which comprise of an index SNP with the highest association and SNPs in high LD with that index SNP. Parameters were chosen to retain SNPs in association with index SNPs with *p* < 0.0001 and *r*^2^ < 0.1 within 3 Mb windows, as used in previous works (Schizophrenia Working Group of the Psychiatric Genomics Consortium, 2014; Xu et al. [34]; Pardiñas et al. [63]). Owing to very high LD within the MHC region, only the most median index SNP and its associated clump were kept from the MHC region. The MHC region was defined as chr6:28,477,797–33,448,354 on the hg19 genome assembly.

The genomic coordinates for disorder-associated loci were found using the index SNPs and the “LD-calculations” procedure on PLINK 1.9 [64]. Data from 1000 Genome’s EUR population (The 1000 Genomes Project Consortium, 2015) was used to remove index SNPs, not in Hardy-Weinberg equilibrium (*p* < 0.0001) or those with a minor allele frequency <0.05. Disorder-associated SNP loci were then defined such that SNPs within loci were associated with index SNPs with *r*^2^ > 0.5 and were within 250 kb of an index SNP. The number of SCZ-associated SNP loci was markedly great than that of BD-associated SNP loci (Supplementary Figure 6). In both disorders, the number of disorder-associated SNP loci is relatively constant for higher *p* value stratifications, decreasing after *p* < 10^−5^.

### Enrichment of nORF-HARs with disorder-associated SNP loci

To determine whether nORFs associated with HARs, especially those DE, are enriched within disorder-associated loci, an enrichment test was performed using INRICH [65]. This was used as it accounts for SNP density as well as overlapping genes (nORFs in this case). The sets of loci used were those generated in the previous section.

The analysis was carried out for the full set of nORF-HARs, as well as the subsets of nORFs associated with vHARs, mHARs, or pHARs. Although INRICH is usually used for the analysis of genes, it can also be used for the analysis of nORFs. INRICH requires four files: an interval file, which contained the loci-defining genomic coordinates for disorder-associated SNP loci and the “rs” IDs of the loci’s index SNPs; an interval map file, which contained the genomic coordinates for and the ‘rs’ IDs of the loci’s index SNPs; a target set file, which contained the genomic coordinates and IDs of the nORF-HARs; and a reference gene file, which contained the genomic coordinates of the 3103 nORFs expressed in the neuropsychiatric samples. Since no SNPs from the GWAS were present on the sex chromosomes, all nORFs on the sex chromosomes were removed before analysis. INRICH merges any overlapping nORFs before processing. Empirical *p* values for enrichment are then calculated through the first round of 5000 permutations. The second round of 5000 permutations corrects for multiple testing and accounts for gene length to give corrected *p* values.

### Identification of unique TEs

In all, 3,987,910 TEs throughout the human genome were identified using RepeatMasker (http://repeatmasker.org/). All coordinates were already based on hg19 genome assembly. TEs that overlapped were merged, resulting in 3,863,891 unique TEs. In-depth analysis to identify unique TEs is best done with the more involved framework developed by Guffanti, G. et al. [49].

### Association of nORFs with TEs

nORFs are defined to be associated with a TE if TE overlapped 2 kb region upstream of the nORF, but not the nORF itself. Association between DE nORF and TEs was investigated to gain insight into the impact of TEs on nearby nORF expression via correlation analysis of expression—this is only useful if TEs not contained in nORFs are examined.

### Identification of DE HARs and DE TEs

A set of transcripts DE between SCZ and BD and controls in the PsychENCODE data sets was identified as mentioned above. HARs and TEs that were included in or overlapped with these DE transcripts were designated DE HARs and DE TEs, respectively. DE TE expression was normalized using the TMM normalization procedure [66] as provided in edgeR v3.30.3 [67].

### Correlation of expression between DE nORFs and their associated DE TEs

Spearman and Pearson correlation coefficients and their corresponding *p* values were calculated for the normalized counts for each DE nORF–DE TE combination (each DE nORF may be associated with many DE TEs). Expression of a DE nORF and its associated DE TE within a DE nORF–DE TE combination was defined to be significantly correlated if the absolute Spearman and Pearson correlation coefficients were above 0.5 and significant (*p* < 0.05) for the DE nORF–DE TE combination.

### Proteogenomic analysis to demonstrate translation of transcribed nORFs

Proteogenomic analysis, as described in Prabakaran et al. [16], Erady et al. [20], and Puntambekar et al. [17], to demonstrate evidence of translation of the transcribed nORFs was performed using 1. amino-acid sequence of all the 248,135 nORFs, 2. transcripts assembled from a subset of PsychENCODE samples, which are part of SMRI Array Collection. For this subset, we had matching raw transcriptomic and proteomic data; however, from different (adjacent) regions of the prefrontal cortex (BA46 and BA10, respectively).

### Analysis of transcripts from SMRI Array Collection samples

RNA-Seq data from BA46 of post-mortem brain samples, classified as Array Collection by SMRI, was obtained upon request [68]. This comprised of 23 SCZ, 23 CNT, and 16 BD samples—after matching with proteomic samples and removing any outliers (Supplementary Figure 7).

In brief, the RNA extraction was performed as follows. 1 μg of total RNA was poly-A selected using oligo-dT Dynabeads, libraries were prepared using Illumina’s TruSeq v1 (Illumina, Hayward, CA) and sequencing was performed using Illumina HiSeq 2000 giving ~3 Mb of 90 bp paired-end reads for each library. The resultant.FASTQ/.FQ files were processed as described below.

The.FASTQ/.FQ was assessed using FastQC for quality control. Read alignment was carried out using HISAT2 v2.1.0 with default parameters except ‘--add-chrname’, ‘—dta’, and ‘--summary-file’ were set to TRUE. Additionally, either Phred +33 or Phred +64 encoding was set to TRUE based on the sample being analyzed. Reads were aligned using the index for GRCh38 genome available at https://ccb.jhu.edu/software/hisat2/manual.shtml. The resultant summary file was used to generate counts of percentage read alignment. (Supplementary Figure 8).

Following alignment, transcripts were assembled using StringTie v1.3.3 (Supplementary Figure 9). First, StringTie was run with default parameters and ‘-A’ set to TRUE to assemble sample-specific transcripts from the aligned reads (.BAM files), using gencode V30 primary comprehensive gene annotation (https://www.gencodegenes.org/human/) as reference. Second, all the.GTF files generated in the previous step were merged using StringTie –merge to create a union transcript data set. Third, StringTie was rerun on the aligned reads with StringTie merged file as the reference and parameters ‘-B’, ‘-e’, and ‘-A’ set to TRUE, allowing us to calculate sample-specific transcript abundances for the union transcript data set. Transcripts were filtered to retain only those from chromosomes 1–22, X, and Y, with TPM > 0.1 in at least 25% samples and no PAR (pseudoautosomal regions) suffix to the transcript IDs. Once again nORFs within this subset of samples were identified using the GffCompare workflow described previously. HISAT2 and StringTie runs were conducted on the cloud server platform provided by Seven Bridges Genomics.

Sample metadata was analyzed for potential confounders using Mann–Whitney *U* pair-wise test for continuous data and Fisher’s test or Chi-square test for categorical data in R. Significance was assigned as ‘*’ for *p* value <0.05, ‘**’ for *p* value <0.01, ‘***’ for *p* value <0.001 and N.S. for non-significant *p* values (Supplementary Figure 10). Additionally, three different batches were identified for the samples, and batch effects were assessed using PCA conducted on expression levels of known genes from chromosomes 1–22, X, and Y with TPM > 0.1 in at least 25% samples. The resultant PCA was then colored based on different metadata categories to identify any metadata-batch relationship (Supplementary Figures 11–13).

To confirm that the gender metadata labeling for our samples was correct, we conducted two analyses. First, using average chromosome Y gene expression levels, samples were split and plotted according to their metadata information to confirm that those annotated females had 0 or low chrY gene expression. Second, hierarchical clustering of XIST gene expression using R functions hclust (method = “single”) and cutree, allowed us to compare the two resultant clusters with their respective gender metadata (Supplementary Figure 14).

### Analysis of mass spectra from SMRI Array collection samples

Details of sample collection, preparation, and LC-MS analysis are described in Gottschalk et al. [55]. In brief, post-mortem anterior prefrontal cortex (BA10) samples were obtained from 23 SCZ, 23 BD, and 23 control samples (after matching with RNA-seq data this led to the use of 23 SCZ, 16 BD, and 23 CNT samples). In all, 50 mg of tissue slices per sample were collected and processed. Protein samples were analyzed using Waters Q-TOF premier mass spectrometer. The output.RAW files were processed on PLGS and converted to.MGF files. The.MGF files were searched against the human UniProt database using Mascot to identify known proteins that are translated.

### Proteogenomics analysis

Unmapped mass spectra were searched against two databases using Mascot. The first search was carried out against nORF amino-acid database that was constructed using 248,135 nORFs that we curated. The second search was performed against a transcript-based nucleotide database assembled using transcripts expressed in all the samples, as described in Prabakaran et al. [16] and Erady et al. [20] (Supplementary Figure 15).

The results of mapping unmatched sample spectra to nORF amino-acid database were filtered by protein and peptide score >50 and expectation value <0.05. Furthermore, only peptides expressed in at least 30% of each disorder group were evaluated (Supplementary Figures 16–18). Expression of the identified nORF proteins was evaluated across different metadata sets namely, gender, psychosis, and suicide. Significant differences in the presence of an nORF protein between the metadata categories were determined using Chi-square goodness of fit test. Significance was determined as * for *p* value < 0.05, ** for *p* value < 0.01, and *** for *p* value < 0.001. A similar analysis was performed for additional novel peptides identified after spectra matching to the transcriptomic database. Additionally, to confirm that the identified peptides are novel, a protein environment was manually curated from the genes in the vicinity of the identified peptide. Finally, each peptide was matched to the curated protein sequences to retain unmapped and unique novel peptides.

### Enrichment analysis to identify potential functions of nORFs

InterProScan was used to identify descriptive GO terms for the nORFs used in this study and GO enrichment was performed using GOEnrichment tool available via usegalaxy.org. Next, using the GLANET tool [61] for annotation and enrichment analysis, DHS1, TFs, and histone modification enrichment were evaluated for nORFs. Default parameters were used and 10,000 samples were processed across 30 core processors.

### Potential structures of identified nORFs

Structures for 21 nORFs that were identified using our proteogenomic analysis, and DE nORFs identified in BD or SCZ, were generated using I-TASSER and Raptor-X as described in Gunnarsson and Prabakaran [18]. Default parameters were used for the structure prediction run. For I-TASSER, the model with the highest confidence score was chosen as the nORF structure. Models were visualized using Avogadro or Jena3D viewer [69].

### Correlation analysis of the translated nORFs with psychosis, suicide, and gender

Expression of the 21 translated nORFs was compared for differences (presence/absence evaluated as yes/no) between gender, the incidence of psychosis, and suicide. Significance was evaluated using a Chi-squared test for each disorder or inter-disorder. *P* value significances were evaluated at three levels: ***<0.001; **<0.01; *<0.05 C. Similarly, the three new nORFs identified using transcriptomic data were compared for differences between gender, the incidence of psychosis, and suicide.

## Results

### Creation of nORF database and classification of nORF entries

In our previous work, we curated ~194,407 nORF entries (nORFs.org) [19]. To this set of nORFs, we added “low-noise” nORFs, as defined and identified across 353 samples from the RPFdbv2.0 using the RibORF tool [52]. Briefly, low-noise nORFs were identified as those with a lower SD of their RPKM read counts to that of the median deviation of canonical ORFs or cORFs (the main ORFs within protein-coding genes). This resulted in 248,135 nORF entries (GRCh38; 247,404 entries in nORF hg19) after removal of nORFs that were in-frame with the cORFs as determined by our classification scheme (Supplementary Figure 2A). These nORF coordinates were then extensively pre-processed to remove duplicates and re-classified based on their genomic locations with respect to known genes.

Classification of the 248,135 nORF entries with respect to known genes (Supplementary Figure 2B) was based on whether they are in-frame or in the alternative frame to their corresponding known protein-coding genes. Approximately, 42% of the nORFs in our data set were identified to be within the CDS of a protein-coding gene, but in an alternative frame. Supplementary Figure 19A displays the number of nORFs localized within known genes classified based on biotypes. Furthermore, we evaluated the potential function of protein-coding genes using FunRich v3.1.3. and genes with nORFs were identified to be associated significantly more with neurological disorders as shown in Supplementary Figure 19B. This analysis indicates that disruption of putative nORF functions could be involved in neuropsychiatric disorders, such as SCZ and BD, which may lead to new diagnostic and therapeutic opportunities.

### Identification of DE nORFs in PsychENCODE data set

**Fig. 1:**
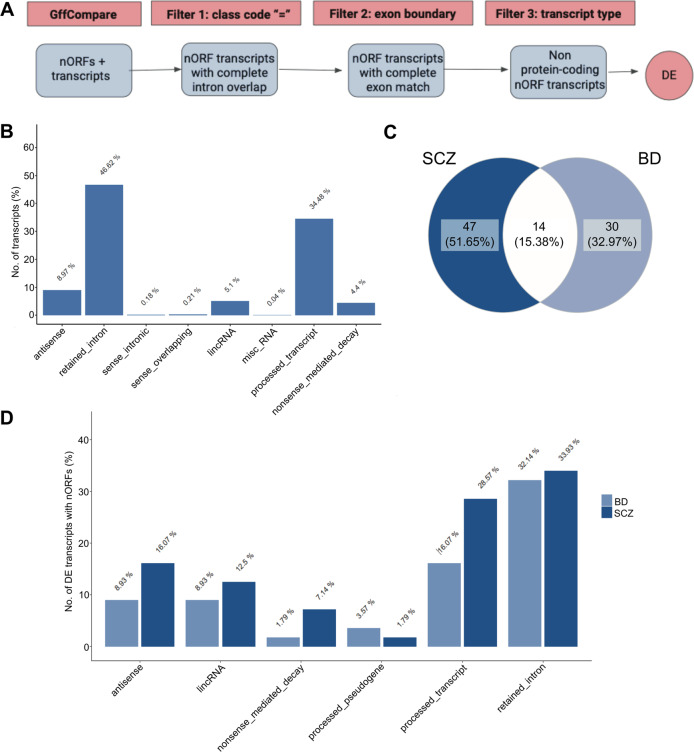
Identification of nORFs within neuropsychiatric samples. **A** The workflow used to identify nORF containing transcripts within neuropsychiatric samples is shown. Genomic coordinate matches between nORFs and sample transcripts assessed using GffCompare, were filtered to retain only matches of the type “=” or complete intron chain overlap. Next, nORFs with exon boundaries contained within their corresponding transcript matches were selected. Finally, only non-coding transcripts, with biotype not equal to “protein-coding”, were retained for further DE analysis. **B** Percentage of the total nORF containing transcripts split over their biotypes shows that ~47% of transcripts are retained introns. **C** The 40 and 56 DE transcripts highlighted in Table 2, contain 44 and 61 nORFs for BD and SCZ, respectively. Venn diagram assessing the 44 BD and 61 SCZ nORFs shows that 14 nORFs are common to and present within DE transcripts in SCZ and BD. **D** For nORFs within DE transcripts, transcript biotype against total transcript percentage is shown. The highest occupancy of nORFs in DE transcripts is within retained introns followed by processed transcripts, similar to **B**.

To investigate whether the 248,135 nORFs that we curated are transcribed in PsychENCODE samples, and whether they are up or downregulated compared to the control samples, we performed the following set of analyses. Transcripts from the three sample groups were pre-processed as discussed in the methods and their abundance was obtained and filtered to retain those with TPM > 0.1 in at least 10% of the samples, resulting in 110,003 transcripts. We identified 3103 nORFs using the workflow illustrated in Fig. 1A, with ~46% within retained introns and ~34% within processed transcripts (Fig. 1B). To identify DE nORFs we used linear mixed-effects models [58] as it accounts for random effects. This analysis revealed that 2935 and 1689 transcripts containing 56 SCZ and 40 BD nORFs, respectively are DE. (Tables 1 and 2). Fourteen DE nORFs were common to both the disorders (Fig. 1C), indicating the overlap of the pathophysiology of the two disorders, and ~30% of the DE nORFs are “retained” introns (Fig. 1D). Supplementary Figure 20 illustrates the location of DE nORFs on all chromosomes.

**Table 1:** Results of the differential expression analysis for SCZ and BD against CNT samples identified using an FDR threshold of 0.05, and the corresponding number of upregulated and downregulated transcripts are presented.

Condition	DE transcripts (FDR < 0.05)	Upregulated transcripts	Downregulated transcripts
BD/CNT	1689	843	846
SCZ/CNT	2935	1263	1672

40/1689 and 56/2935 transcripts identified as DE in BD and SCZ, respectively, contain nORFs.

**Table 2:** Table summarizes the number of nORFs contained within DE transcripts identified in neuropsychiatric samples.

Condition	DE transcripts containing nORFs	Upregulated transcripts	Downregulated transcripts
BD/CNT	40/1689	21	19
SCZ/CNT	56/2935	25	31

40/1689 and 56/2935 transcripts identified as DE in BD and SCZ, respectively, contain nORFs.

**Fig. 2:**
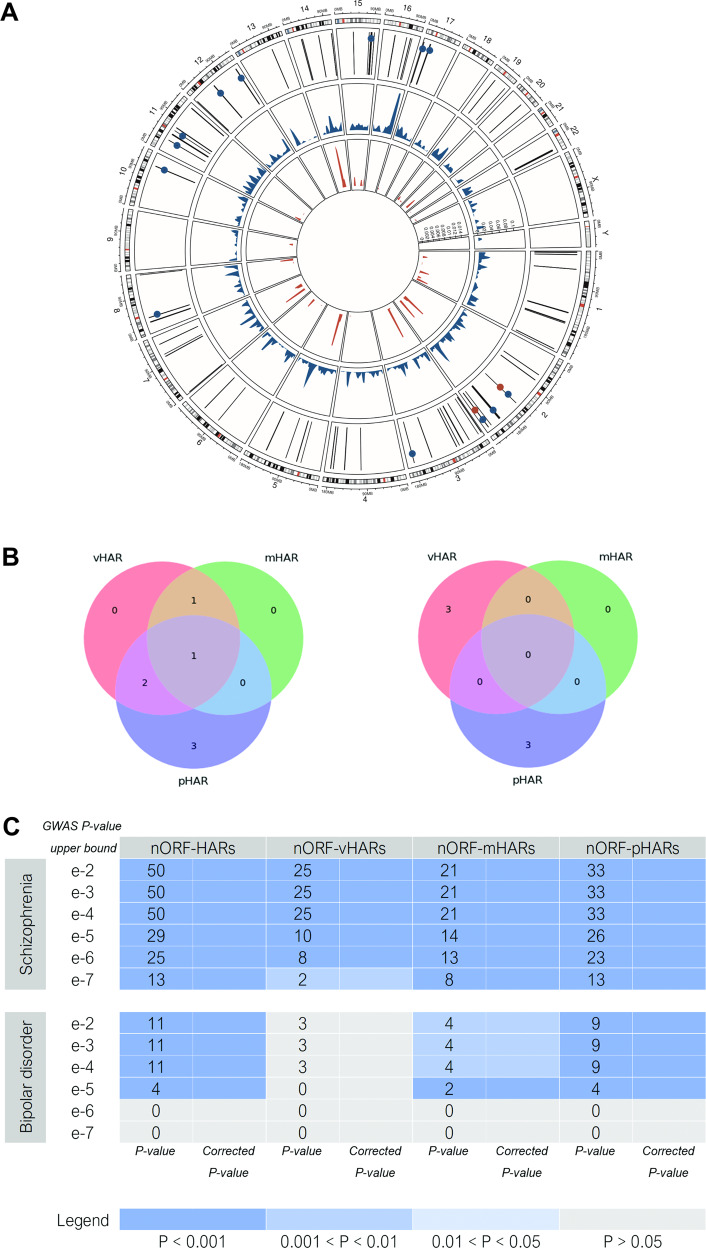
nORF enrichment within HARs and SCZ-specific loci. **A** nORFs transcribed within neuropsychiatric samples (blue peaks) and DE in SCZ (red peaks), were evaluated for overlap and enrichment within SCZ- specific loci (black vertical lines—outermost circular panel), using GLANET. If nORF enrichment was identified, the corresponding loci (vertical line) is marked with circular dots—transcribed nORF enrichment as blue circles and DE nORF enrichment as red circles. Two SCZ loci within chromosome 2 are interesting as they are enriched for nORFs DE in SCZ. Similar analyses conducted for BD-specific loci and SCZ-specific CNVs (copy-number variations) showed no nORF enrichment (Supplementary Figure 21). **B** nORFs transcribed within neuropsychiatric samples and DE in SCZ (left) and BD (right) were defined to be associated with a unique HAR if the unique HAR overlapped the nORF or regions extending 100 kb upstream or downstream of the nORF. For each HAR-associated nORF, each unique HAR with which the nORF was associated was categorized based on the types of HARs—vHARs, mHARs, and pHARs—contained within it. Since a unique HAR could contain multiple individual HARs, a single unique HAR could be categorized as containing multiple types of HARs. The number of unique HARs in each category was quantified. **C** nORFs transcribed within neuropsychiatric samples were defined to be associated with a unique HAR if the unique HAR overlapped the nORF or regions extending 100 kb upstream or downstream of the nORF. Disorder-associated SNPs were stratified based on their genome-wide association study *P* value (‘GWAS *P* value upper bound’). Stratified SNPs were used to determine stratified disorder-associated SNP loci. HAR-associated nORFs (nORF-HARs) were queried for enrichment with stratified disorder-associated SNP loci, using INRICH. The enrichment analysis was also performed for nORFs associated with vHARs (nORF-vHARs), mHARs (nORF-mHARs), or pHARs (nORF-pHARs). The enrichment analysis provided both an empirical *P* value (‘*P* value’) and a *P* value corrected for multiple testing (‘Corrected *P* value’). Both values were categorized based on the indicated limits to produce a heatmap. For each set of stratified disorder-associated loci and each set of nORF-HARs, the number of nORFs that overlapped a locus was quantified and is displayed in the relevant cell in the heatmap.

As we demonstrated in the case of cancer in Erady et al. [20], that differential expression of some nORFs significantly correlate with the survival of patients and hence might be associated with the disease pathology, we intended to investigate similar relationships between differential expression of nORFs in SCZ and BD and their association with the respective disease pathology. Because there is no equivalent metric to patient survival, we explored whether the identified DE nORFs, in the respective disorders, are associated with already identified genomic “hotspots” for the respective disorders. To do this, we used GLANET, a program that associates nORFs with genomic loci that are implicated in SCZ and BD, and tests for the statistical significance of the enrichments. Figure 2A and Supplementary Figure 21 display the results of this analysis as circular plots. If nORF enrichment was identified, the corresponding loci (vertical line) is marked with circular dots—enrichment for nORFs that are transcribed is depicted as blue circles, and enrichment for nORFs that are DE is depicted as red circles. It is interesting to note that two SCZ loci within chromosome 2 (Fig. 2A) are enriched for nORFs DE in SCZ. Similar analyses conducted for BD-specific loci and SCZ-specific CNVs (copy-number variations) showed no nORF enrichment (Supplementary Figure 21).

### nORFs-HARs and their enrichments within disorder-associated SNP loci

Having demonstrated that some nORFs are indeed associated with SCZ hotspots, we performed the following analysis to investigate whether the nORFs constitute recently evolved vHARs, mHARs, and pHARs genomic regions. Out of 3103 nORFs, 431 nORFs overlapped with 4481 unique HARs (compiled as described in the methods section).

Seven nORFs DE in SCZ (three overexpressed and four underexpressed) were found to be associated with HARs (seven DE nORF-HARs) (Supplementary Table 4); most associated HARs resided within the same characterized region as their nORF, but some were found in intergenic regions or in different genes (Supplementary Table 4). Six nORFs DE in BD (four overexpressed and two underexpressed in BD) were found to be associated with HARs (six DE nORF-HARs) (Supplementary Table 4); again, most associated HARs resided within the same characterized region as their nORF, but some were found in intergenic regions or in different genes.

The transcript types of the seven DE nORF-HARs in SCZ are −2 “antisense”, 2 “processed transcripts”, 1 “nonsense-mediated decay”, 1 “retained intron”, and 1 “lincRNA”. Two DE nORFs contained HARs within them: tracer_65443 and fs1rH2. The transcript types of the six DE nORF-HARs in BD are −3 “retained intron”, 1 “lincRNA”, 1 “processed pseudogene”, and 1 “antisense”. No nORFs contained HARs within their lengths. The HAR types associated with DE nORFs in SCZ and The HAR types associated with DE nORFs in BD are displayed in Fig. 2B (left and right panel, respectively).

INRICH analysis revealed that out of the 431 nORF-HARs, 50 are associated with SCZ loci with a GWAS *p* value upper bound of 10^−2^; 13 nORF-pHARs were associated with SCZ loci with a GWAS *p* value upper bound of 10^−7^. Furthermore, 11 nORF-HARs are associated with BD loci with a GWAS *p* value upper bound of 10^−2^, and only four nORF-pHARs were associated with BD loci with a GWAS *p* value upper bound of 10^−5^ (Fig. 2C). The DE nORF tracer_65443 and its parent gene ZEB2 were both within an SCZ-associated locus (SNP locus that involved SNPs with *p* value 10^−7^ < *P* < 10^−6^). The parent gene (*SCL7A6OS*) of one DE nORF (tracer_42939) was within an SCZ-associated locus *p* < 10^−7^ and was also associated with a BD-associated SNP locus that involved SNPs with 10^−5^ < *P* < 10^−4^. This is consistent with phenotypic overlap between the two disorders as well as recent findings that the two disorders share some susceptibility genes, suggesting some commonality in the causes behind the two disorders. The association of DE nORF-pHARs enriched in SCZ loci suggests that these DE nORFs and their functions may have arisen in primates and then been subject to increased evolution in the human lineage, only to result in SCZ susceptibility in modern humans when dysfunctional.

If nORFs are defined to be associated with a HAR if the HAR overlapped the nORF or regions extending 1 kb upstream or downstream of the nORF, we identify 54 nORF-HARs. Of these 54 nORF-HARs, two were DE in SCZ: fs1rH2 and tracer_65443. None were DE in BD (Supplementary Table 5). Nine of these 54 nORF-HARs are associated with SCZ loci to a GWAS *p* value upper bound of 10^−2^, and 6 are associated with SCZ loci to a GWAS *p* value upper bound of 10^−5^. Unlike the analysis previously done, there is little difference in SCZ-associated SNP enrichment between nORF-vHARs, nORF-mHARs, and nORF-pHARs. None of these 54 nORF-HARs is enriched in BD-associated SNPs (Supplementary Figure 22).

### DE HARs and DE TEs

HARs and TEs that were included in or overlapped with these DE transcripts were designated as DE HARs or DE HARs and DE TEs, or DE TEs, respectively. In all, 160 DE transcripts in SCZ contained HARs resulting in 305 DE HARs in SCZ; 59 DE transcripts in BD contained HARs resulting in 90 DE HARs in BD; 2638 DE transcripts in SCZ contained TEs resulting in 176,100 TEs DE in SCZ; and 1522 DE transcripts in BD contained TEs, giving 93,717 TEs DE in BD.

### Association of DE nORFs with DE HARs (DE HARs)

While most HARs are considered non-coding genomic regions, they do demonstrate evidence of transcription. RNAs containing HARs fall under various classifications of non-coding RNA—sRNA, miRNA, lncRNA, or eRNA—or may simply be a part of a known protein-coding region. If a DE HAR associated with a DE nORF is within a known protein-coding region, that could indicate a potential connection between that protein-coding region and the DE nORF. Three DE nORFs were found to be associated with DE HARs in SCZ (3 DE nORF–DE HARs); none were found in BD (Supplementary Figure 23, Supplementary Table 6). As the set of transcripts used to identify DE HARs was also used to identify the DE nORFs, the two DE nORFs that contain HARs within their lengths were identified as DE nORF- DE HARs. The third DE nORF- DE HAR had its DE HAR in a gene different to the DE nORF (tracer_87517) (Supplementary Table 6). None of the three DE nORF–DE HAR were within SCZ-associated loci.

### Association of DE nORFs with DE TEs (DE TEs)

The presence of a TE in the 2 kb region upstream of a DE nORF could indicate the presence of an alternative promoter. Therefore, DE nORFs were investigated for association with DE TEs based on the condition that DE nORFs are associated with a DE TE if the TE is within the 2 kb region upstream of the nORF. Eleven DE nORFs were found to be associated with DE TEs in SCZ (11 DE nORF- DE TEs), and eight DE nORFs were found to be associated with DE TEs in BD (eight DE nORF- DE TEs). Of the eight DE nORFs associated with DE TEs in BD, two are also associated with HARs: cp2xH1 and eveeH1. DE TEs could allow for different expressions of nORFs under different conditions, leading to phenotypes of SCZ or BD. Besides differential expression-based regulation we also investigated whether there could be other unknown correlations between the expression of TEs and nORFs. To understand this, we performed Spearman and Pearson correlation analysis of the expression of nORFs and each of their associated DE TEs.

Table 3 displays significantly correlated DE nORF–DE TE combinations in SCZ and BD, with the details of the TE type, class, and clade, as found on Dfam [70]. Five DE nORF–DE TE combinations had significantly correlated expression levels in SCZ (more details of these DE TE combinations can be found in Supplementary Table 7). Notably, the DE nORF 2vnjH1 had its expression significantly correlated with two DE TEs: one 3′-end-of-L2 LINE and one L2-end SINE. One DE nORF was overexpressed in SCZ; 4 DE nORFs were underexpressed in SCZ. The DE nORFs’ biotypes were split into: two “lincRNA”, two “processed transcripts”, and one “antisense”. None of the DE nORFs were within SCZ-associated loci. The 5 DE TEs were all unique and were comprised of 2 3′-end-of-a-L2 LINEs, 2 L2-end SINEs, and one 3′-end-of-a-L1 LINE. For BD, four DE nORF–DE TE combinations were found to have significantly correlated expressions (Table 3 & Supplementary Table 7). Of the four DE nORFs, two were found to be associated with HARs as well. The four DE nORFs’ biotypes were split evenly between “retained intron” and “lincRNA”. None of the DE nORFs were within BD-associated loci. The four DE TEs were also all unique and were comprised of two ERV1 LTRs, one Alu SINE, and one 3′-end-of-a-L1 LINE. Three DE nORFs were upregulated in BD; one DE nORF was downregulated. The DE nORFs included cp2xH1 and eveeH1, which were also associated with HARs, suggesting that those DE nORFs were under HAR-related selection pressure as well as being regulated by TEs. This association is perhaps most significant for eveeH1 and is interesting given the parent gene of eveeH1 is ZNF84, a zinc finger protein that contains a KRAB/FPB domain [71] that may regulate gene expression through TE regulation [72]. As such, eveeH1 may serve as an initial regulation point from which other TE-associated genes and nORFs may be regulated. Its associated DE TE with correlated expression is an endogenous retrovirus sequence ERV1 conserved in primates; its insertion may have conferred an added layer of regulation that was later selected for along with the associated HAR, perhaps in part owing to its far-reaching effects. One DE nORF–DE TE combination with significantly correlated expression was shared between the SCZ and BD data sets: tracer_18675 with its L1MC2 TE. It is not surprising that the expression of the DE TE was correlated with the expression of the DE nORF, since there is a significant overlap between the DE TE and the DE nORF. This was the only DE nORF–DE TE combination with significant overlap between the DE TE and the DE nORF.

**Table 3:** Significantly correlated DE nORF–DE TE combinations in SCZ and BD, detailing the TE type, class, and clade, as found on Dfam [70].

	nORF	TE	TE Type (clades)
SCZ	jsksH2	chr21: 16135328: 16135426: L2a	3′ end of L2. *LINE*. (Theria)
	2vnjH1	chr1: 212870591: 212870745: MIRb	L2-end. *SINE*. (Mammalia)
		chr1: 212871710: 212871835: L2a	3′ end of L2. *LINE*. (Theria)
	tracer_18675	chr11: 69240406: 69240975: L1MC2	3′ end of L1. *LINE*. (Eutheria)
	geqkH1	chr15: 61056439: 61056636: MIR3	L2-end. *SINE*. (Mammalia)
BD	cp2xH1	chr9: 44401599: 44401814: MER90a	ERV1. *LTR*. (Eutheria)
	96nxH6	chr5: 141523284: 141523556: AluJo	Alu. *SINE*. (Primates)
	eveeH1	chr12: 133624216: 133624355: MER65A	ERV1. *LTR*. (Primates)
	tracer_18675	chr11: 69240406: 62940975: L1MC2	3′ end of L1. *LINE*. (Eutheria)

More detailed information can be found in Supplementary Table 6.

### Translation evidence of nORFs in brain samples

Although we showed direct evidence of nORF transcription in SCZ and BD PsychENCODE samples and even though these nORFs have evidence of translation from other studies, we aimed to obtain direct evidence of translation of these nORFs in SCZ and BD brain samples. To this end, we used a proteogenomic approach that combines both transcriptomic and proteomic data as discussed in Prabakaran et al. [16] and Erady et al. [20]. Because mass spectrometry data for the PsychENCODE samples were not available, we performed the proteogenomics analysis only on a subset of samples for which both transcriptomic and proteomic data were available. However, these data had certain limitations. Although the transcriptomic and proteomic data were obtained from the same patients, the samples were collected from adjacent yet slightly different brain regions. In addition, the proteomics data were obtained using a mass spectrometry instrument with moderate sensitivity that does not cover the entire proteome. Despite these limitations, the data were still suitable for investigating the potential translation of nORFs. Transcriptomic and proteomic data from this subset of 62 samples from the SMRI Array cohort was analyzed using the proteogenomic framework as described in the methods and displayed in Supplementary Figure 15.

**Fig. 3:**
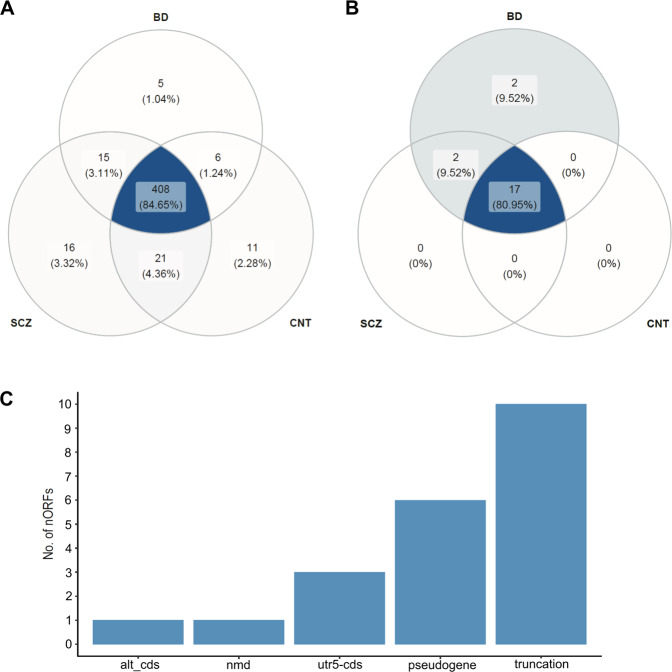
Translated nORFs in neuropsychiatric samples. **A** 482 known proteins were identified across CNT, SCZ, and BD samples upon proteomic analysis, of which 408 were common to all three, and 11, 16, and 5 proteins were unique to CNT, SCZ, and BD samples, respectively. **B** 21 nORFs were identified as translated of which 17 were common between CNT, SCZ, and BD. In addition, two nORFs were unique to BD and two to SCZ and BD. **C** Translated nORFs were split according to their annotation type-identified with reference to the transcripts within which the nORFs are contained. 10/21 nORFs are truncations of the main transcript and 6/21 are within pseudogenes.

The proteogenomic analysis identified 446, 460, and 434 known proteins that were translated in CNT, SCZ, and BD, respectively, among these 408 is common between all three sample sets (Fig. 3A). The results were filtered to retain entries with a peptide expectation score <0.05 and a peptide score >50, which were expressed in at least 30% of samples from each of the three groups. Additionally, each peptide entry that passed the filtration criteria was evaluated manually for novelty by matching against all known protein fragments. As a result, 21 nORFs from our curated list of 248,135 nORFs were identified as translated along with three novel ones, which were identified from the transcriptomic data. However, these three novel peptides were identified within four of the 21 nORFs. The number and identity of the peptides that mapped to the nORFs are listed in Supplementary Table 8.

Seventeen of the 21 nORFs identified as translated were common between CNT, SCZ, and BD whereas two were unique to BD and two to SCZ and BD (Fig. 3B, Supplementary Figure 24). Ten out of the 21 nORF proteins were annotated as truncations of known proteins and six as pseudogenes (Fig. 3C). nORFs uniquely expressed in SCZ or BD (two common to SCZ and BD and two unique to BD) were present within genes such as syntaxin (a presynaptic membrane protein) binding protein (STXBP1), heat shock protein (HSPA2), and DISC1 fusion partner (DISC1FP1), some of them are associated with SCZ and BD. We found one nORF- ajg1H1, that had evidence of both transcription and translation and contained a tubulin domain as determined using InterProScan.

**Fig. 4:**
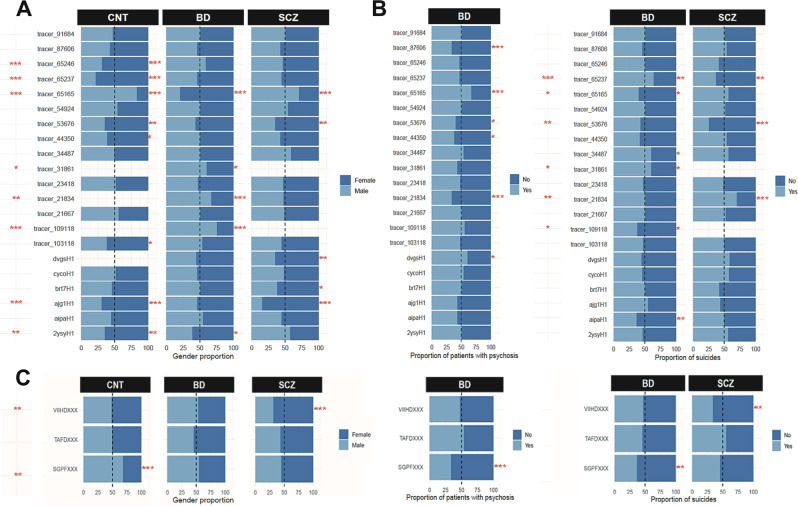
Metadata-specific differences in translated nORFs. **A**, **B** Expression of the 21 translated nORFs were compared for differences (presence/absence evaluated as yes/no) between **A** gender, **B** incidence of psychosis and suicide. Significance was evaluated using a Chi-squared test for each disorder (right of each bar) or inter-disorder (to the left of the nORF ids). *p* value significances: ***<0.001; **<0.01; *<0.05. **C** Similarly, three unique novel peptides identified via proteogenomic analysis were compared for differences between gender, incidence of psychosis and suicide. Novel peptides with significant differences between the metadata categories evaluated using Chi-squared test were identified at *p* value significances: ***<0.001; **<0.01; *<0.05.

We further evaluated the expression differences of these novel peptides between disorders for metadata categories such as suicide, psychosis, and gender and identified significant expression differences as determined using Chi-squared tests (Fig. 4). We found that eight of the 21 nORFs were significantly associated with gender, six of the 21 nORF were significantly associated with psychosis in BD, and six of the 21 nORFs were significantly associated with suicide in SCZ and BD. Among the three additional nORFs peptides, two were significantly different between the genders, one was significantly associated with psychosis, and two were significantly associated with suicide. This analysis revealed that if such nORF expression and their disruptions are manifested in peripheral tissues of SCZ or BD patients as well, we can potentially develop diagnostic strategies to stratify or diagnose patients who might develop psychosis or who might be prone to suicide based on their expression.

### GO enrichment analysis for potential functional inferences of nORFs

To infer functions of the translated nORFs from their amino-acid sequence we performed GO analysis. For all the 248,135 nORFs used in this study, GO terms were obtained using InterProScan and GO term enrichment was performed using GOEnrichment tool via the galaxy server (Supplementary Figure 25). For the 3103 nORFs with evidence of transcription, structural molecular activity within ribosomes, and therefore, potential involvement in translation was found. For nORFs that were DE, no enrichment was found, possibly owing to the small set of DE nORFs with GO terms (2 in BD and 13 in SCZ). nORFs identified as translated within our samples showed enrichment for structural molecular activity as part of the myelin sheath and cytoskeleton, GTP binding, GTPase, and other oxidoreductase activity.

The GLANET analysis, in addition to associating nORFs with the SCZ and BD disorder-associated loci, also identified enrichment of certain DHS1, histone modifications, and TFs within the transcribed and DE nORFs (Supplementary Figure 26).

### Potential structures of identified nORFs

**Fig. 5:**
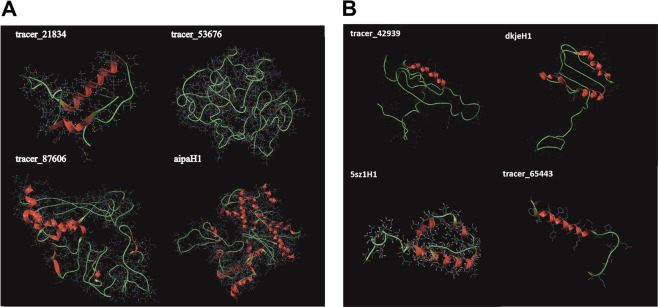
Structure prediction for translated nORFs. **A** Structures were predicted for the 21 translated nORFs, 4 of which are shown here along with their nORF ids (top-left). These nORFs were found to be significantly different in BD and SCZ patients for psychosis or suicide. **B** Example of predicted structures for nORFs that are DE in BD (up–top-left; down–top-right) and SCZ (up–bottom-left; down–bottom-right) and were found to be associated with HARs. Additionally, the DE nORF tracer_65443 and its parent gene ZEB2 were both within an SCZ-associated locus (SNP locus that involved SNPs with *p* value 10^−7^ < *P* < 10^−6^). The parent gene (SCL7A6OS) of one DE nORF (tracer_42939) was within an SCZ-associated locus *p* < 10^−7^ and was also associated with a BD-associated SNP locus that involved SNPs with 10^−5^ < *P* < 10^−4^.

To infer whether these nORFs could form potential structures as we did previously in Erady et al. [20] and Gunnarsson and Prabakaran, 2021 [61] we predicted the putative structures of the 21 nORFs identified as translated, as well as DE nORFs that included nORFs that were associated with pHARs and present in SCZ loci, using I-TASSER and Raptor-X. For I-TASSER, the model with the highest confidence score was chosen as the nORF structure. Figure 5 shows representative structures of four nORFs out of the 21 nORFs for which we had evidence of translation and representative nORF structures for those associated with pHAR and SCZ, and BD loci. All other remaining structures are displayed in Supplementary Figure 27.

The results of this analysis provide a basis for many avenues of future work. Using the structures proposed here, potential functions could be queried. The cellular role of these nORF protein products could be interrogated by analyzing their interactomes. We have also embarked on preliminary studies to investigate whether nORF protein products could be targeted by small molecules, in case their disruptions could be firmly established as causation for these two disorders.

## Discussion

The lack of adequate and targetable SCZ and BD-specific signatures in protein-coding and non-coding genes, led us to investigate nORFs within the human genome as we did previously in Erady et al. [20]. We curated 248,135 nORFs and investigated 1340 neuropsychiatric samples from the PsychENCODE consortium and identified 3103 nORFs as transcribed, with 56 and 40 nORFs DE in SCZ and BD, respectively. In addition, DHS1, TF, and histone modification enrichments were found within the transcribed nORFs, and SCZ-specific loci were found enriched with transcribed and DE nORFs.

A number of nORFs DE in SCZ and BD were identified as being associated with HARs and as having their expression correlated with that of associated TEs DE in SCZ and BD. The association of 13 DE nORFs with HARs, especially those that are also associated with SCZ and BD loci, suggests that HARs may play a role in the pathophysiology of SCZ and BD, and that these DE nORFs may have advantageous functions that they have been selected for either as a result of or in tandem with their associated HARs. For example, it could be possible that HARs are crucial in regulating gene expression in certain stages of neurodevelopment—perhaps this is the reason why they have been selected for over time—but at a later point in time, their erroneous activity, which could be stimulated by environmental agents, leads to susceptibility to or development of SCZ or BD. This may contribute to explaining how environmental factors impact the development of neuropsychiatric disorders. This also reinforces the idea that susceptibility genes for the two disorders may have been positively selected for in human-specific evolution.

The type of HAR associated with each DE nORF gives a glimpse into the evolutionary background of their regulatory relationships and of, by extension, the disorders in question. The depletion of vHARs in DE nORF-HARs with respect to pHARs and mHARs in the SCZ data sets (Supplementary Figure 23) reinforces past conclusions that pHAR- and mHAR-associated genes (and therefore nORFs) are under greater selective constraint than vHAR-associated genes [34]. The same cannot be said for DE nORF-HARs in the BD data sets.

The results of the enrichment analysis (Fig. 2C) reveal that for SCZ more HAR-associated nORFs show significant enrichment with the imputed regions until nominal *P* < 10^−7^ except the results for vHAR-associated nORFs, and for BD, HAR-associated nORFs as a whole are less significantly enriched with disorder-linked loci, and none of the vHAR-associated nORFs show significant enrichment. These results show that nORFs-HARs may not only play a role in SCZ as shown by Xu et al. [34] but also in BD although to a lesser extent. More importantly, SCZ loci are strongly enriched in nORFs near the pHARs. The nORFs fs1rH2 and tracer_70164, which are DE in SCZ, have functions indicated by past work to localize at post-synapses and the postsynaptic density [73, 74]. The DE nORF tracer_65443, which is DE in SCZ, is a retained intron within *ZEB2*. *ZEB2* is a DNA-binding transcriptional corepressor that binds to E-boxes. It is involved in the transforming growth factor-beta signaling pathway [75] and is largely found in tissues derived from the neural crest: many symptoms of ZEB2 deficiency can be explained by aberrant development of the neural crest-derived structures [76]. It is highly conserved throughout evolution [76], and the *ZEB2*-associated DE nORF tracer_65443 is associated with many HARs, of all three conservation backgrounds. The DE nORF tracer_65443 and *ZEB2* are also within an SCZ-associated locus, illustrating the importance of both the DE nORF and the gene in SCZ. In addition, evidence from recent literature demonstrates that ZEB2 promotes neuroepithelial transition, and its manipulation and downstream signaling leads to the acquisition of non-human ape architecture in the human context and vice versa, establishing an important role for neuroepithelial cell shape in human brain expansion [77].

Similar patterns can be written for nORFs DE in BD. The DE nORF tracer_42939 is within the *SLC7A6OS* gene, which is highly conserved in vertebrates and has been shown to play a critical role in zebrafish central nervous system (CNS) development. Despite the gene’s conservation in vertebrates, it is associated with a pHAR, suggesting that some event may have occurred around the divergence of primates that resulted in the human-lineage-specific rapid evolution of that locus, possibly resulting in altered CNS development and susceptibility to BD. As mentioned previously, *SLC7A6OS* is within an SCZ-associated locus; its detection as a DE nORF-HAR in BD and its relevance to SCZ suggests a genetic commonality and may contribute towards explaining phenotypic similarities between the disorders.

The correlation of expression of DE nORFs and DE TEs indicates the possibility of TE-based regulation of the DE nORFs, especially since the majority of DE TEs found in this analysis are completely distinct from the DE nORF (the exception is tracer_18675 and its L1MC2 TE). A particularly interesting DE nORF–DE TE combination is that of eveeH1 and its ERV1 LTR TE. The DE nORF eveeH1 is within the *ZNF84* gene, which codes for a KRAB/FPB domain-containing protein [71]. The KRAB/FPB domain may regulate gene expression through TE regulation [72]; eveeH1 may thus be an initial regulation point from which a cascade of TE-based regulation occurs. As it is differentially regulated in BD, it could be responsible, at least in part, for TE-based differential regulation across the genome that contributes to the BD phenotype. Further investigation into the specific function of eveeH1 and other DE nORFs may elucidate more fully their role in SCZ and BD. How the differential regulation arises is a matter of future interest—a possible explanation is that the ERV1 LTR TE, whose transcriptional regulation of eveeH1 and *ZNF84* may be crucial for certain stages of neurodevelopment, is stimulated to act erroneously by environmental factors at a later point of time, potentially drawing a parallel between the involvement of TEs and HARs in these disorders.

Although literature evidence suggests that TEs might have an independent evolutionary role in SCZ and BD [28, 42, 48, 49] besides regulating the expression of nORFs and HARs as we have shown, the exact relationships between HARs, TEs, and nORFs remain to be elucidated; further work utilizing ChIP-seq and whole-genome bisulfite sequencing data could shine a light on them. Furthermore, analysis of more RNA-seq data—from a large number of disorder samples, in particular—would help clarify how HARs and TEs regulate nORF expression in these two mental disorders.

We also demonstrated evidence of translation for 21 nORFs from our database, and for three new ones identified from the transcriptome of a smaller subset of neuropsychiatric samples. The major limitation of these findings is the lack of appropriate MS data, as the SMRI subset the transcriptomic and MS data were obtained from a slightly different brain region of the same patients. Of the 21 nORFs, some were found significantly different between disorders for metadata categories such as gender, the incidence of psychosis, and suicide. We predicted structures for the 21 nORFs and for those that are associated with pHARs and disorder-loci. Our approach could offer a new strategy to expedite the identification of novel drug candidates and novel diagnostic signatures for preemptive interventions, for example, to prevent suicide or mitigate psychosis.

To summarize, we introduce how novel regions of the genome, nORFs, merit systematic analysis within disease systems to uncover novel targets for the development of diagnostic and therapeutic strategies. From an evolutionary point-of-view, as we have shown in the cichlids fishes adaptive radiation that nORFs in accelerated regions may have a role to play in speciation and fitness [17], our current results indicate that the genomic features responsible for SCZ and BD arose at least after the divergence of mammals from other vertebrates, or that nORFs associated with pHARs may have arisen in primates and then been subject to increased evolution in the human-lineage, only to result in SCZ and, to a lesser extent, BD susceptibility in modern humans when dysfunctional. It may be that these newly emerged genomic features are the ones that are more easily disrupted due to environmental perturbations resulting in the disease pathogenesis than the older “fixed” ones. More work has to be done to evaluate this claim.

## Supplementary information

Supplementary Figures

Supplementary Table 1

Supplementary Table 2

Supplementary Table 3

Supplementary Table 4

Supplementary Table 5

Supplementary Table 6

Supplementary Table 7

Supplementary Table 8

## Data Availability

Codes for this work can be obtained from https://github.com/PrabakaranGroup/norfs_in_neuropsychiatric_disorders.
