# Supplementary material for: Novel open reading frames in human accelerated regions and transposable elements reveal new leads to understand schizophrenia and bipolar disorder
Source: Mol Psychiatry. 2021 Dec 23;27(3):1455–68. doi: 10.1038/s41380-021-01405-6 (PMC9095477; doi:10.1038/s41380-021-01405-6)
Supplement: Supplementary file 1 — Supplementary Figures [file 41380_2021_1405_MOESM1_ESM.pdf]

Supplementary Materials

Novel Open Reading Frames in Human Accelerated Regions and Transposable Elements reveal new leads to understand schizophrenia and bipolar disorder

Chaitanya Erady [1†], Krishna Amin [1†], Temiloluwa O. A. E. Onilogbo [1,2], Jakub Tomasik [2], Rebekah Jukes-Jones [3], Yagnesh Umrania [4], Sabine Bahn [2], and Sudhakaran Prabakaran [5*]

[1] Department of Genetics, University of Cambridge, CB2 3EH, UK

[2]Department of Chemical Engineering and Biotechnology, University of Cambridge, Cambridge, UK

[3] Leicester Cancer Research Centre, RKCSB, University of Leicester, University Road, Leicester, LE1 7RH, UK

[4]Cambridge Centre for Proteomics, Department of Biochemistry, University of Cambridge, Tennis Court Road, Cambridge CB2 1QR, UK

[5]NonExomics, Inc, 2 Simon Willard Road, Acton, MA 01720, US

[†]These authors contributed equally to the manuscript

*Corresponding author, email: sudhakaran.prabakaran@nonexomics.com

Supplementary Figure 1

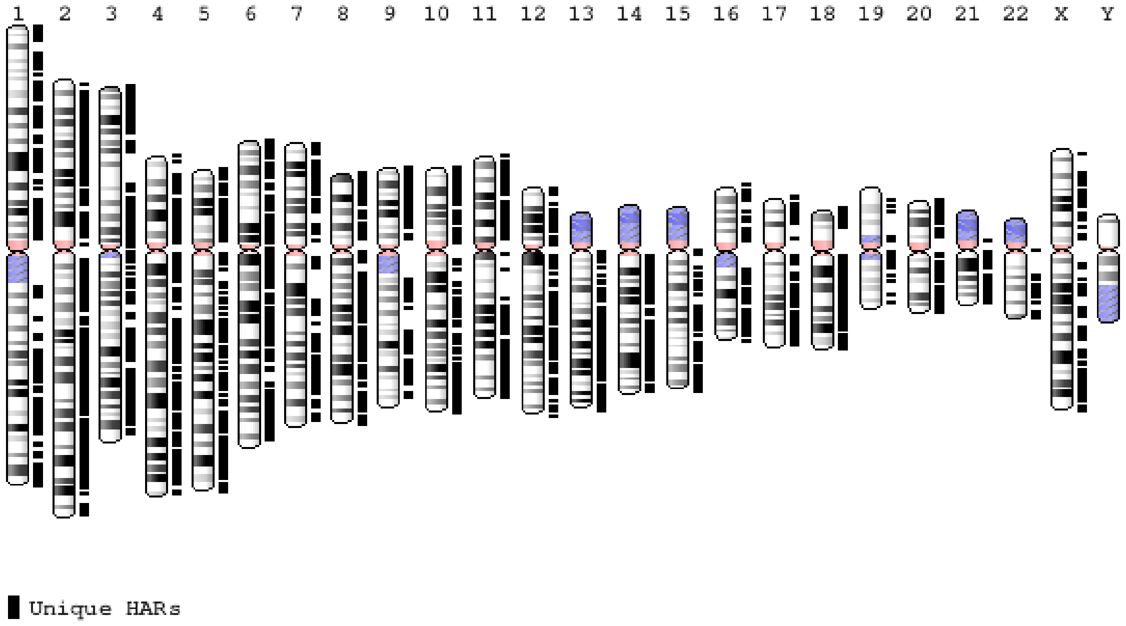

Supplementary Figure 1. An ideogram of unique HARs. Constructed using https://www.ncbi.nlm.nih.gov/genome/tools/gdp) the figure illustrates the location of unique HARs within the hg19 genome assembly.

Supplementary Figure 2

Supplementary Figure 2A

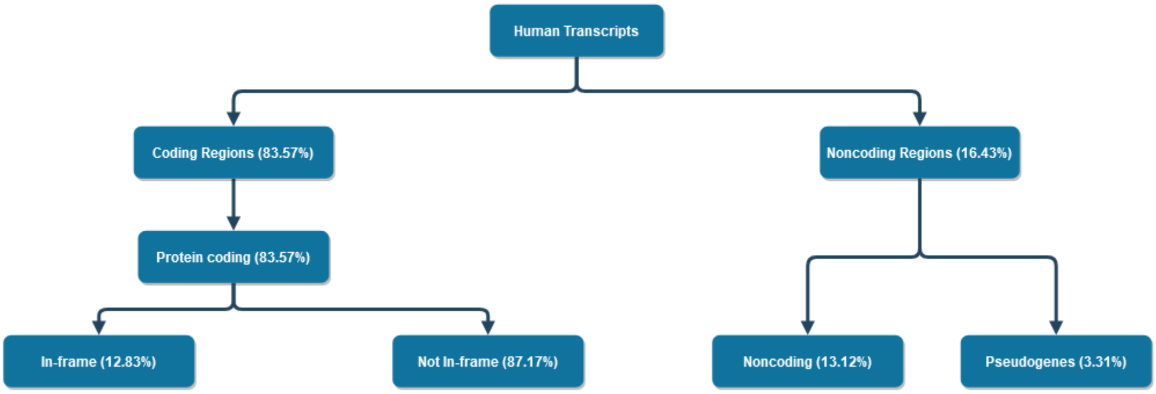

Supplementary Figure 2B

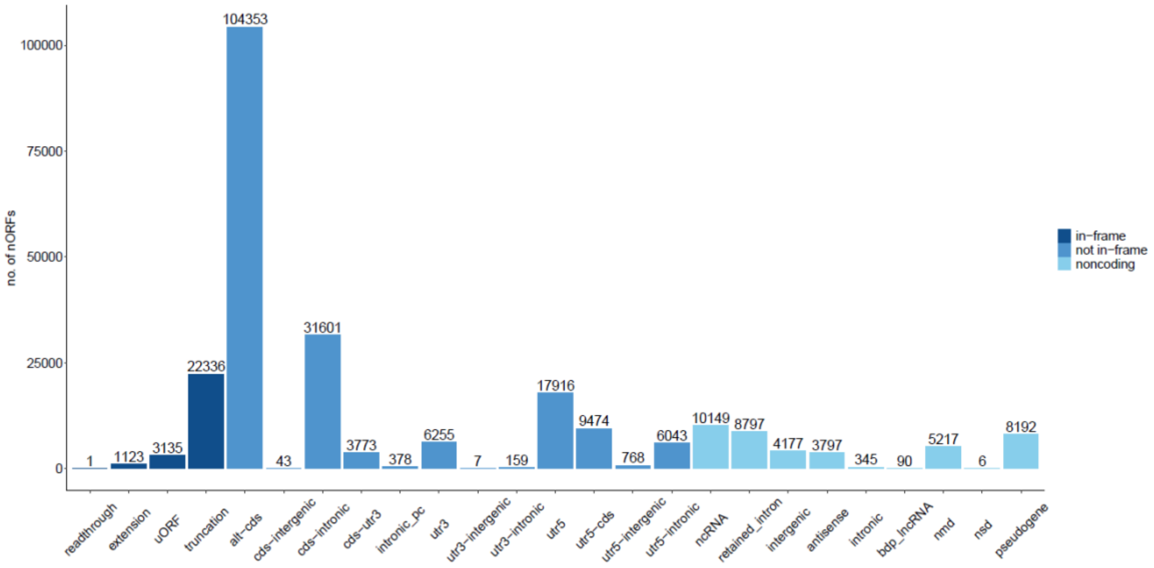

Supplementary Figure 2. Classification of nORFs. A. The 248,135 curated nORF entries were classified with respect to known genes. The translation frame was compared between nORFs and protein-coding genes and the nORF was categorised as in-frame or not in-frame. For not in-frame nORFs, their genomic position in relation to the gene structure was annotated. For nORFs in noncoding region, the overlapping transcripts' biotype was used for categorisation. **B.** Approximately, 42% of the nORFs in our dataset are within the CDS of a protein-coding gene, albeit in an alternative frame.

Supplementary Figure 3

Supplementary Figure 3A

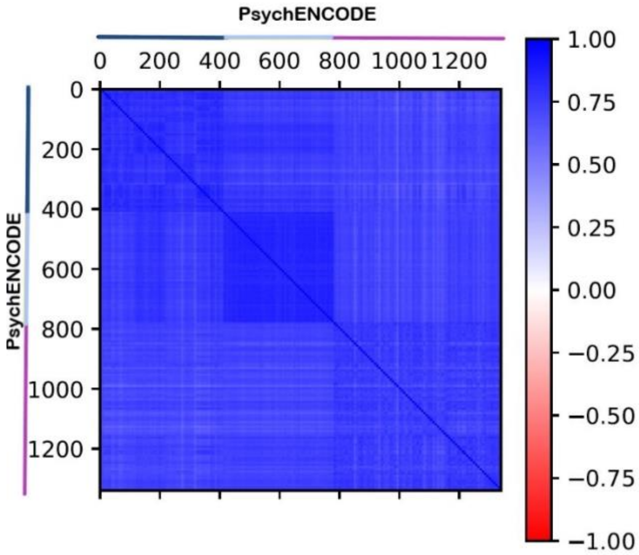

Supplementary Figure 3B

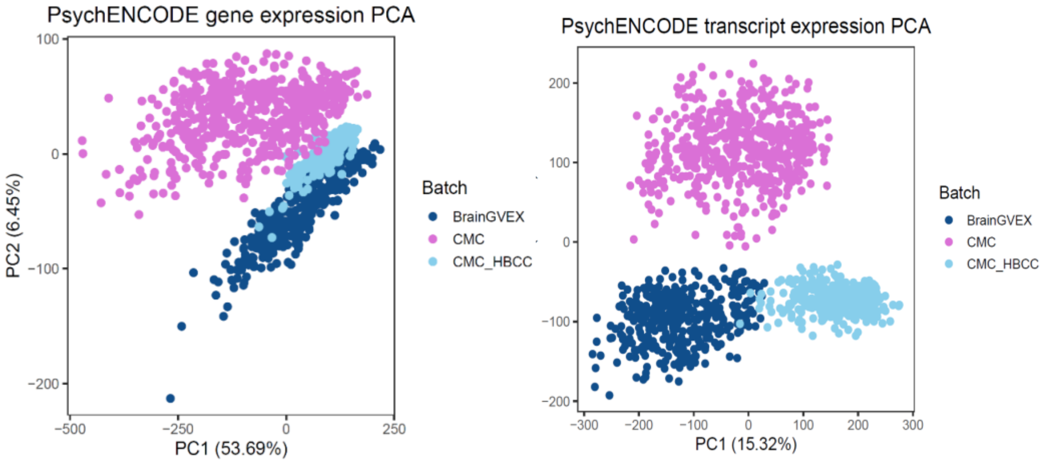

Supplementary Figure 3. PsychENCODE sample set. A. Three different studies from the PsychENCODE consortium were used in our analysis. Transcript expression correlation (Spearman's correlation) between the different samples analyzed in this study shows greater correlation between samples from the same study than those from different studies. Dark Blue cluster – BrainGVEX; Light Blue cluster – CMC_HBCC; Pink cluster – CMC. **B.** PCA of gene and transcript expression for the samples highlights the gene-level and transcript-level differences based on the study used. SVA and MARS analysis were utilized for additional covariate identification for downstream DE analysis.

Supplementary Figure 4

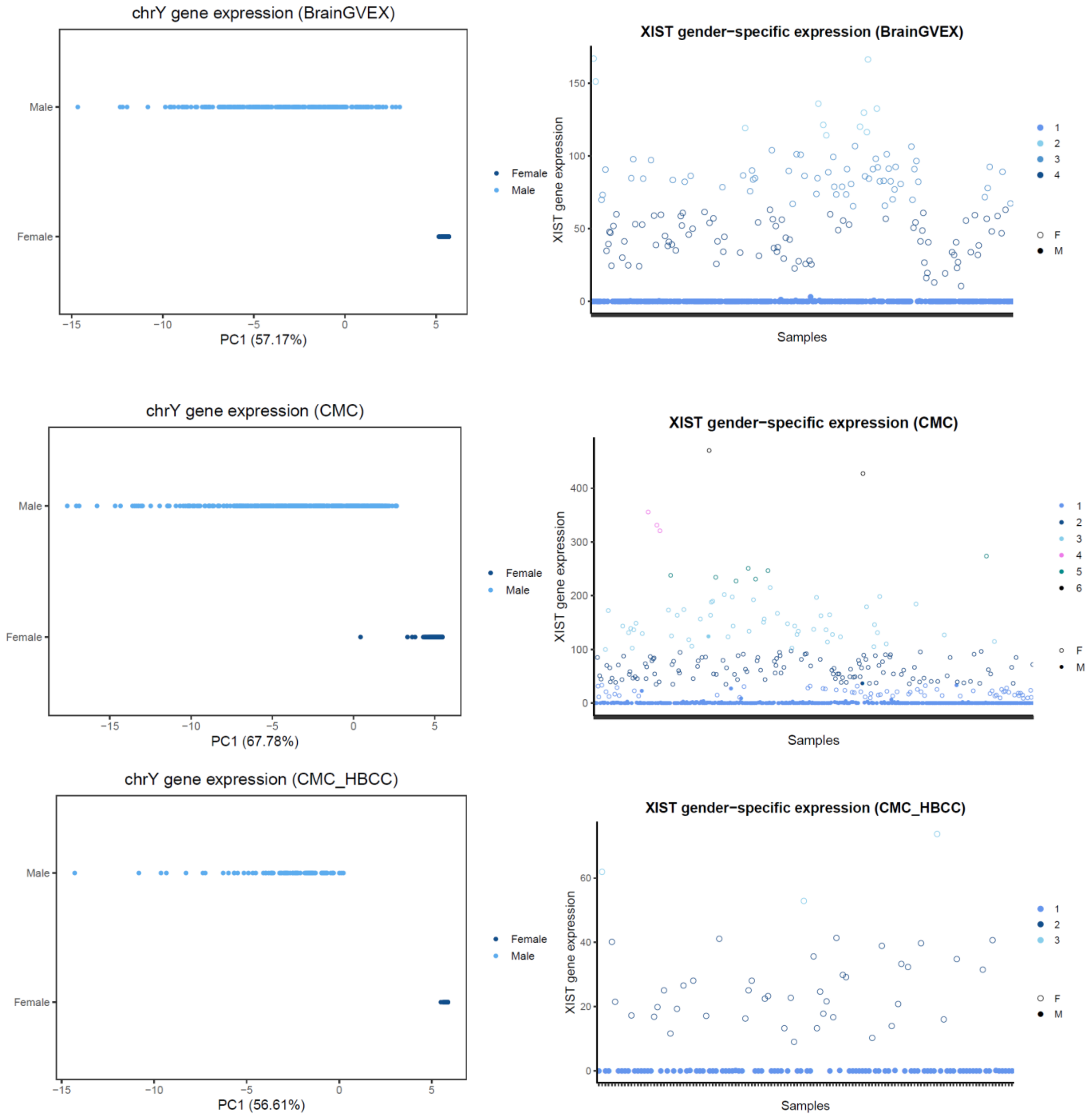

Supplementary Figure 4. Metadata gender labels evaluation against gene expression for PsychENCODE dataset. Male and female metadata sample labels were evaluated by comparing chrY (left) and XIST (right) gene expression for three different PsychENCODE studies – BrainGVEX (top), CMC (middle) and CMC_HBCC (bottom). PCA based clustering of the chrY gene expression

split on the y-axis based on the original metadata labels, shows no discrepancy in sample labelling for BrainGVEX and CMC_HBCC, whereas the male and female clusters overlap for CMC. Hierarchical clustering of XIST gene expression gave 3 clusters coloured with different shades of blue. Open circles represent female samples and closed circles represent male samples. XIST expression analysis did not identify any sample labelling discrepancy for BrainGVEX and CMC_HBCC. XIST expression analysis on the CMC study, revealed female samples within the lower XIST expression cluster and a few male samples within the higher XIST expression clusters.

Supplementary Figure 5

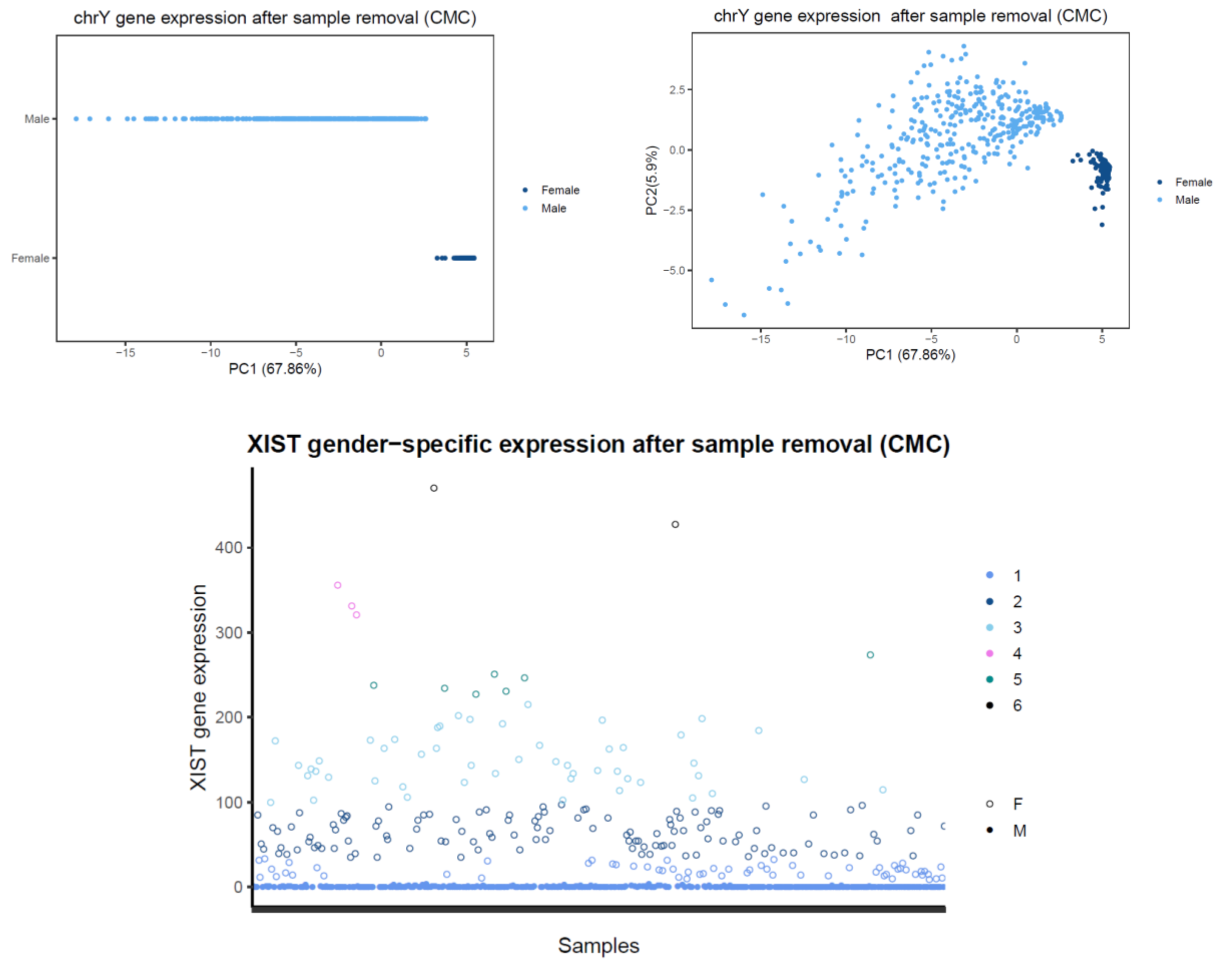

Supplementary Figure 5. Metadata gender labels evaluation against gene expression for PsychENCODE study: CMC. Male and female metadata sample labels evaluated by comparing chrY and XIST gene expression revealed potential incorrect labelling for a few samples within the CMC study (supplementary figure 19). After removing these seven samples, chrY (top) and XIST (bottom) expression was re-evaluated. PCA of chrY expression plotted across top 2 PCs (top right) confirmed two distinct male and female clusters. No further sample label discrepancies were identified

Supplementary Figure 6

	$P < 10^{-2}$	$P < 10^{-3}$	$P < 10^{-4}$	$P < 10^{-5}$	$P < 10^{-6}$	$P < 10^{-7}$
SCZ	1407	1407	1404	672	347	181
BD	455	455	455	151	62	24

Supplementary Figure 6: The number of disorder-associated SNP loci for each disorder and each p-value cut-off.

Supplementary Figure 7

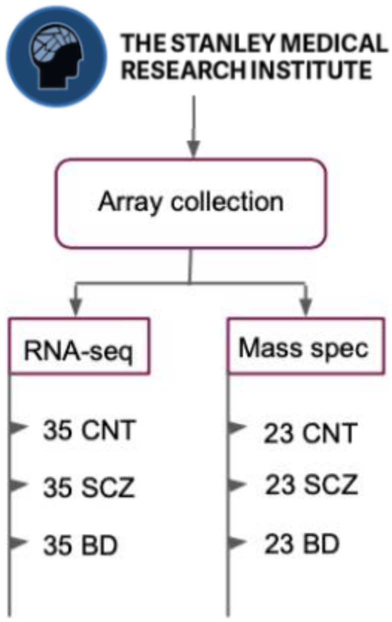

Supplementary Figure 7. SMRI samples used in our analysis. RNA-seq and Mass spec samples for CNT, SCZ and BD were obtained from the Array Collection via SMRI. Available sample numbers are highlighted in the figure.

Supplementary Figure 8

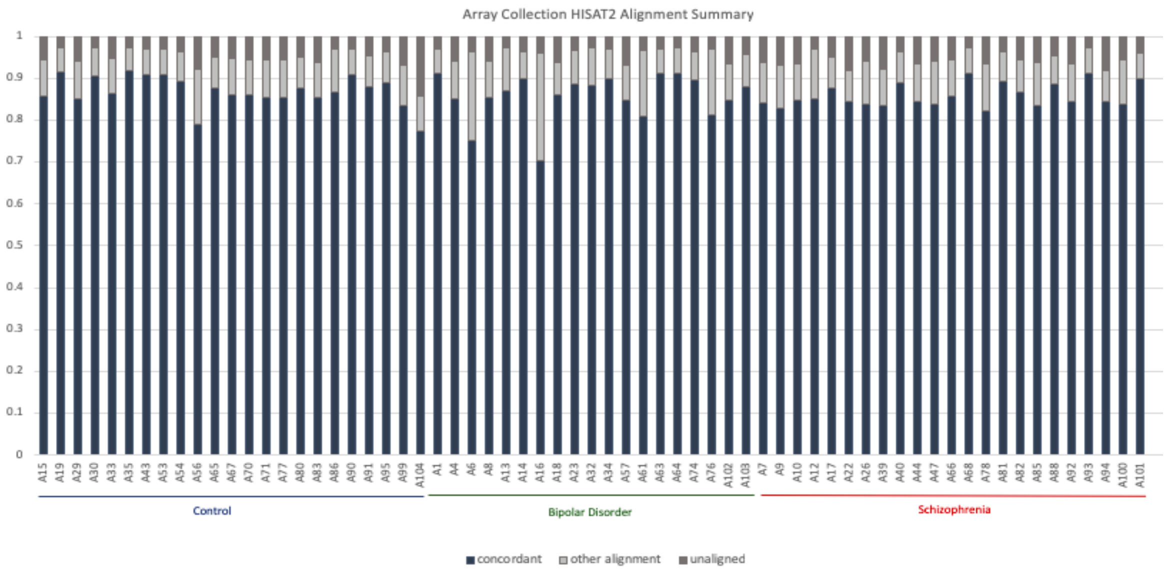

Supplementary Figure 8. HISAT2 alignment of SMRI samples. HISAT2 alignment summary showing ~95% alignment rate. Y-axis shows proportion of different types of read alignments. Concordant alignment (dark blue) refers to reads that aligned to the reference genome with a specified orientation (forward-reverse) and within a specific distance with respect to each other. Other alignment (light grey) contains reads that mapped concordantly >1-time, discordant alignments as well as single reads of a mate pair that aligned at least 1 time. Unaligned (dark grey) refers to reads that aligned to the genome 0 times.

Supplementary Figure 9

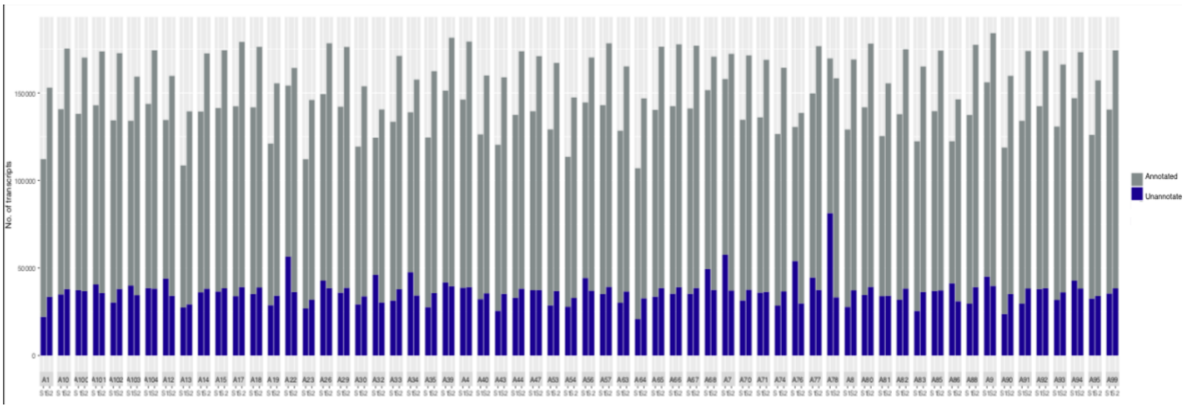

Supplementary Figure 9. StringTie transcript assembly of SMRI samples. No. of transcripts identified before and after StringTie merge. Blue bars indicate unannotated transcripts (no corresponding ENSMEBL identifiers), and grey bars indicate transcripts with Ensembl ids.

Supplementary Figure 10

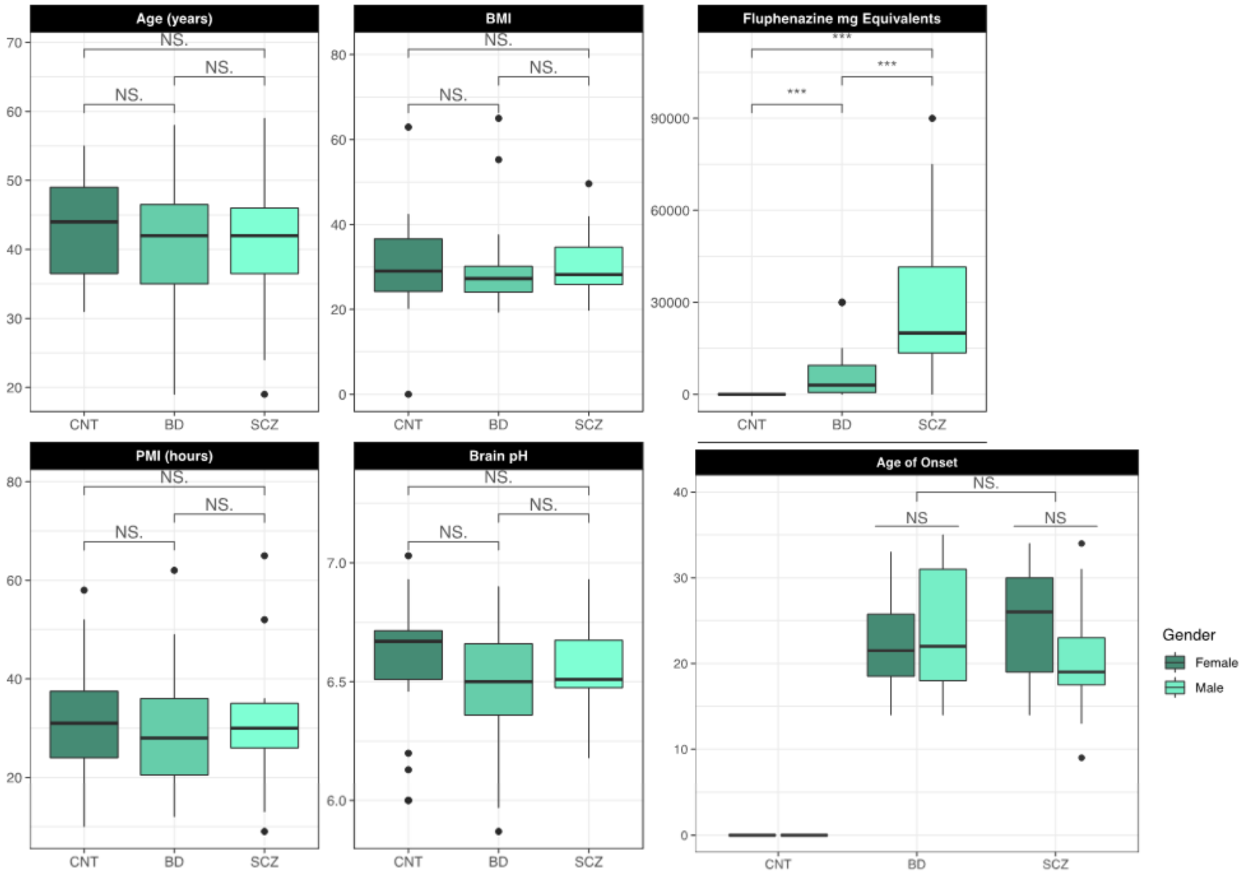

Supplementary Figure 10. Identification of confounding factors using sample metadata. Significant differences between disorder and control for each metadata category was evaluated using Mann-Whitney U test in R. Significance was assigned as '*' for p-value < 0.05, '**' for p-value < 0.01, '***' for p-value < 0.001 and N.S. for non-significant p-values. Fluphenazine concentration was considered as a covariate for downstream DE analysis.

Supplementary Figure 11

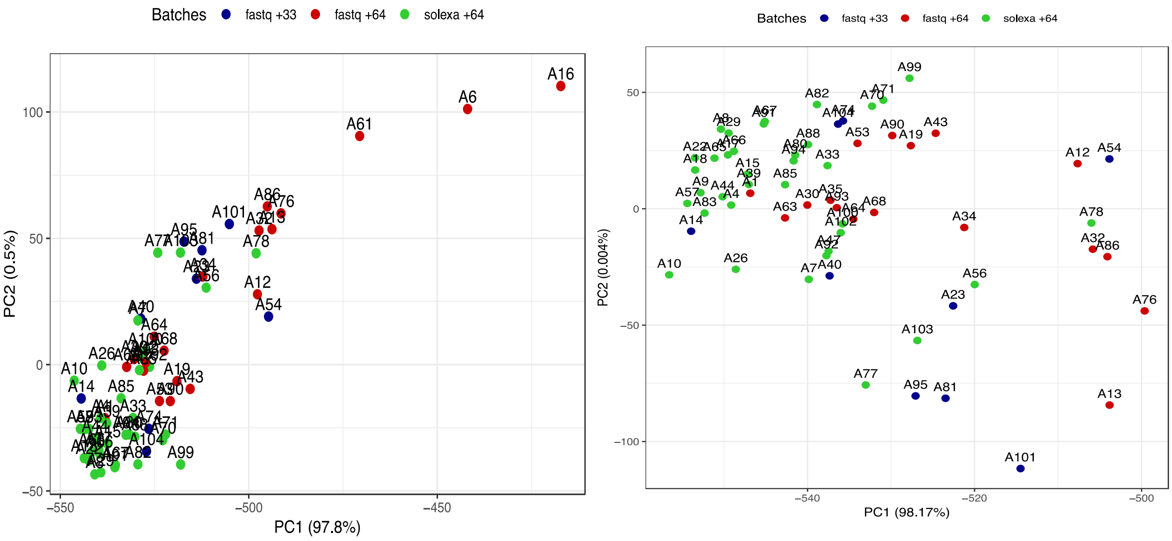

Supplementary Figure 11. PCA of gene expression levels for Array samples before and after outlier sample removal. After conduction a PCA analysis on gene expression values of our samples, three outlier samples: A61, A6 and A16 (left plot) were removed and the recalculated gene expression levels were replotted (right plot). Labels denote sample ids. The three batches identified were fastq +33: FASTQ files with Phred +33 encoding; fastq +64: FASTQ files with Phred +64 encoding; solexa +64: FASTQ files with +64 encoding and file names suffixed with ‘solexa’.

Supplementary Figure 12

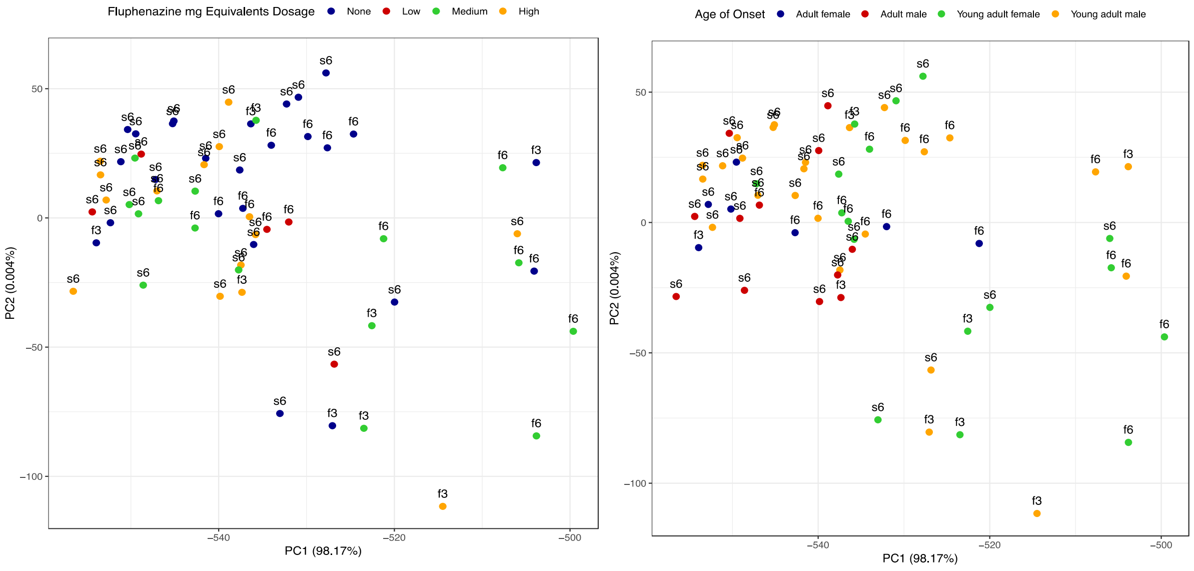

Supplementary Figure 12. PCA re-done after sample removal evaluated for batch-metadata correlations. 17 different sample metadata categories were evaluated for batch-metadata category correlation. Different colours indicate different sub-categories being evaluated and labels represent different batches.

Supplementary Figure 13

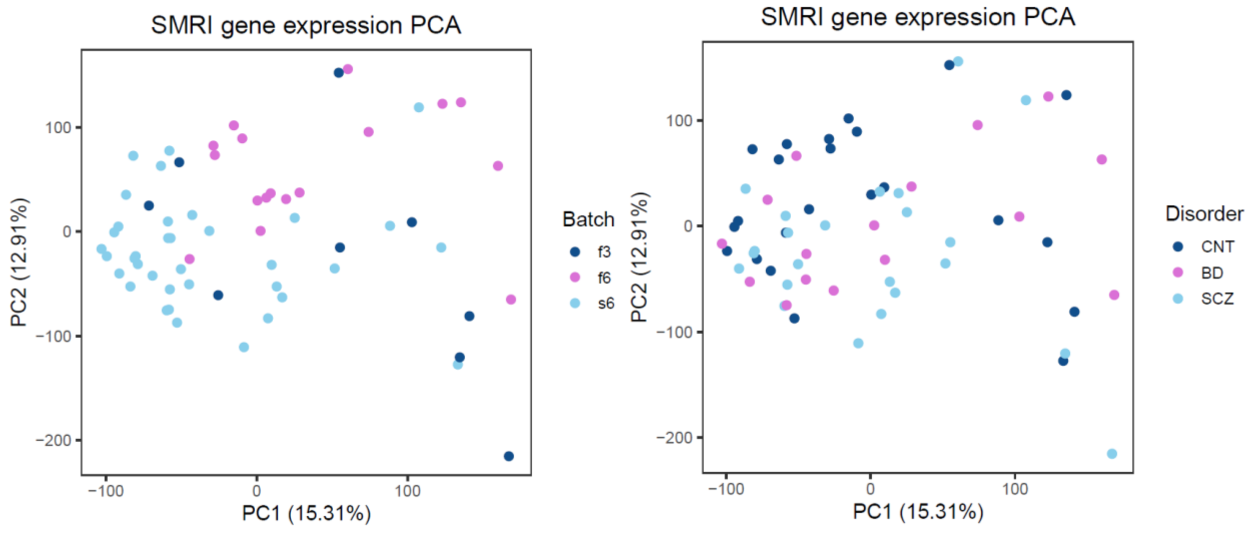

Supplementary Figure 13. Gene expression PCA of SMRI samples. Gene expression PCA of SMRI samples based on batch (left) and disorder (right) is shown in the figure above.

Supplementary Figure 14

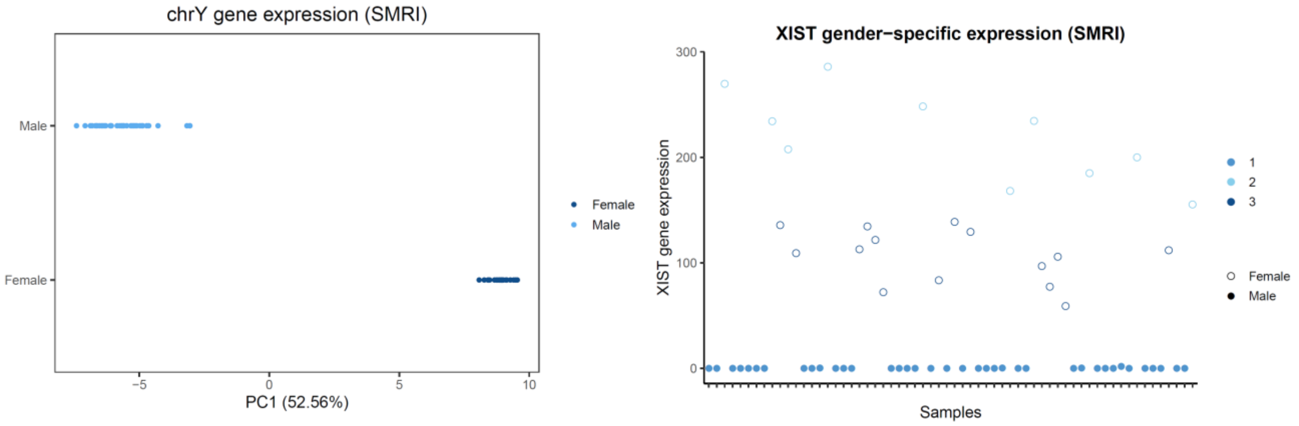

Supplementary Figure 14. Metadata gender labels evaluation against gene expression for SMRI dataset. Male and female metadata sample labels were evaluated by comparing chrY (left) and XIST (right) gene expression. PCA based clustering of the chrY gene expression split on the y-axis based on the original metadata labels, shows no discrepancy in sample labelling. Hierarchical clustering of XIST gene expression gave 3 clusters coloured with different shades of blue. Open circles represent female samples and closed circles represent male samples. XIST expression analysis did not identify any sample labelling discrepancy either.

Supplementary Figure 15

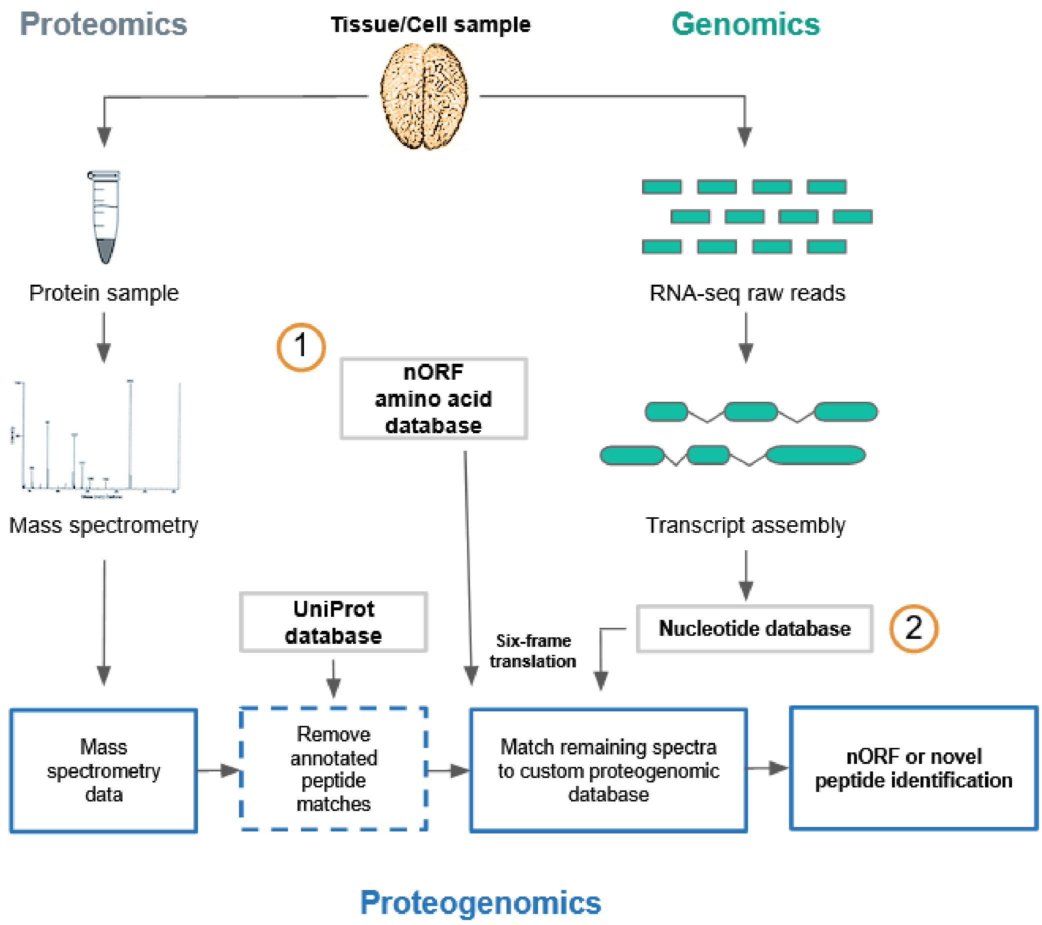

Supplementary Figure 15. Proteogenomic workflow for novel peptide identification. Proteomic samples collected from brain tissue were processed using mass spectrometry, and known proteins were filtered out based on matches of the resultant spectra against the human UniProt database. Any unmapped spectra were processed in two ways: (1) by mapping against the amino acid sequences of nORFs and (2) by processing matched RNA-seq samples isolated from the same brain tissue, assembling the transcripts and six-frame translating their sequences to map against the MS spectra. After additional filtration steps, this proteogenomic workflow allows for the identification of novel peptides or nORF peptides within our protein samples.

Supplementary Figure 16

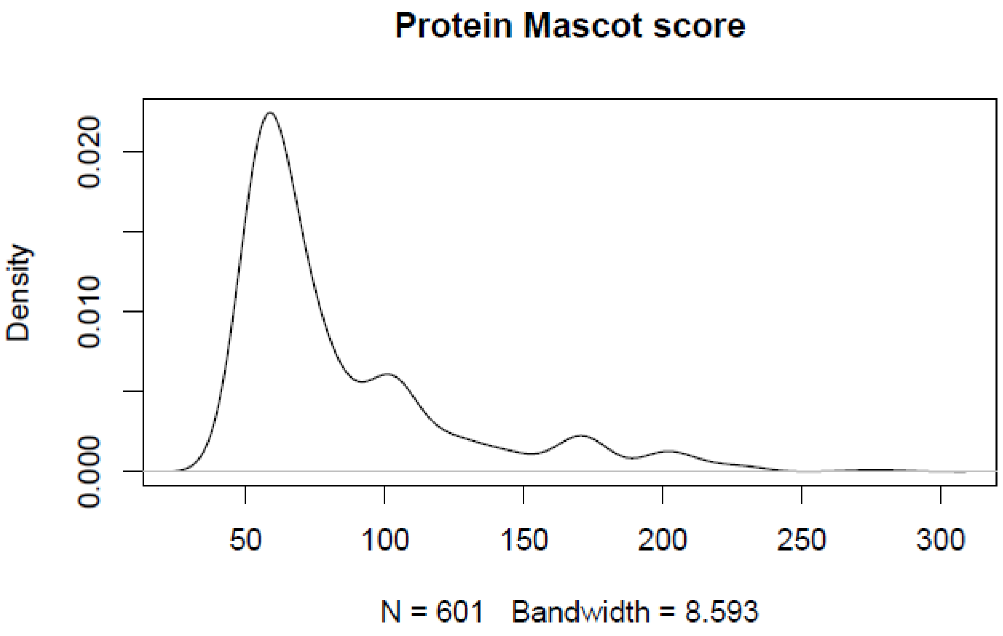

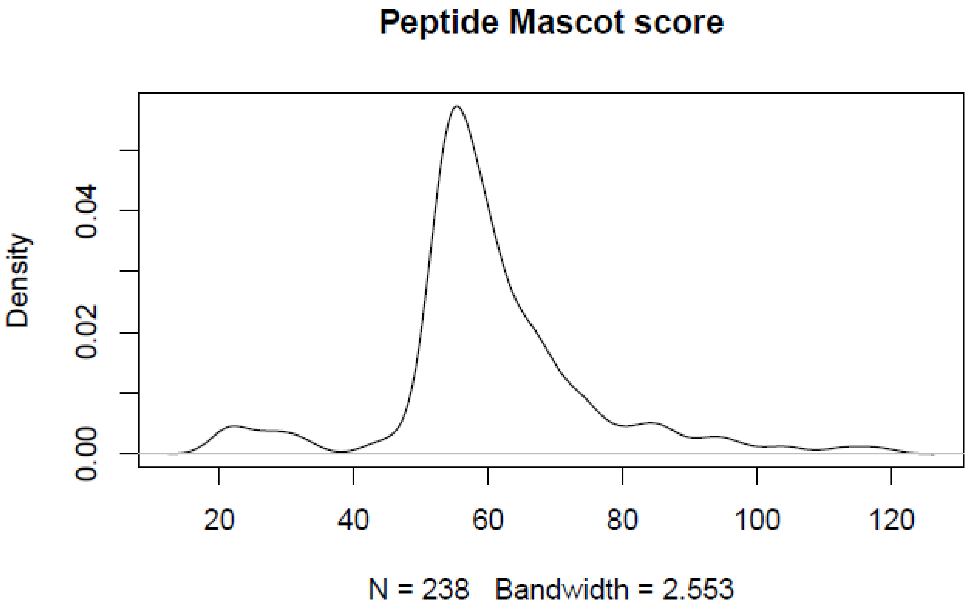

Supplementary Figure 16. Novel peptide identification. Distribution of MASCOT peptide and protein scores calculated for novel peptides in neuropsychiatric samples using proteogenomic analysis are shown. For novel peptide filtration, a peptide score of greater than 50 and peptide expectation value of less than 0.05 was used.

Supplementary Figure 17

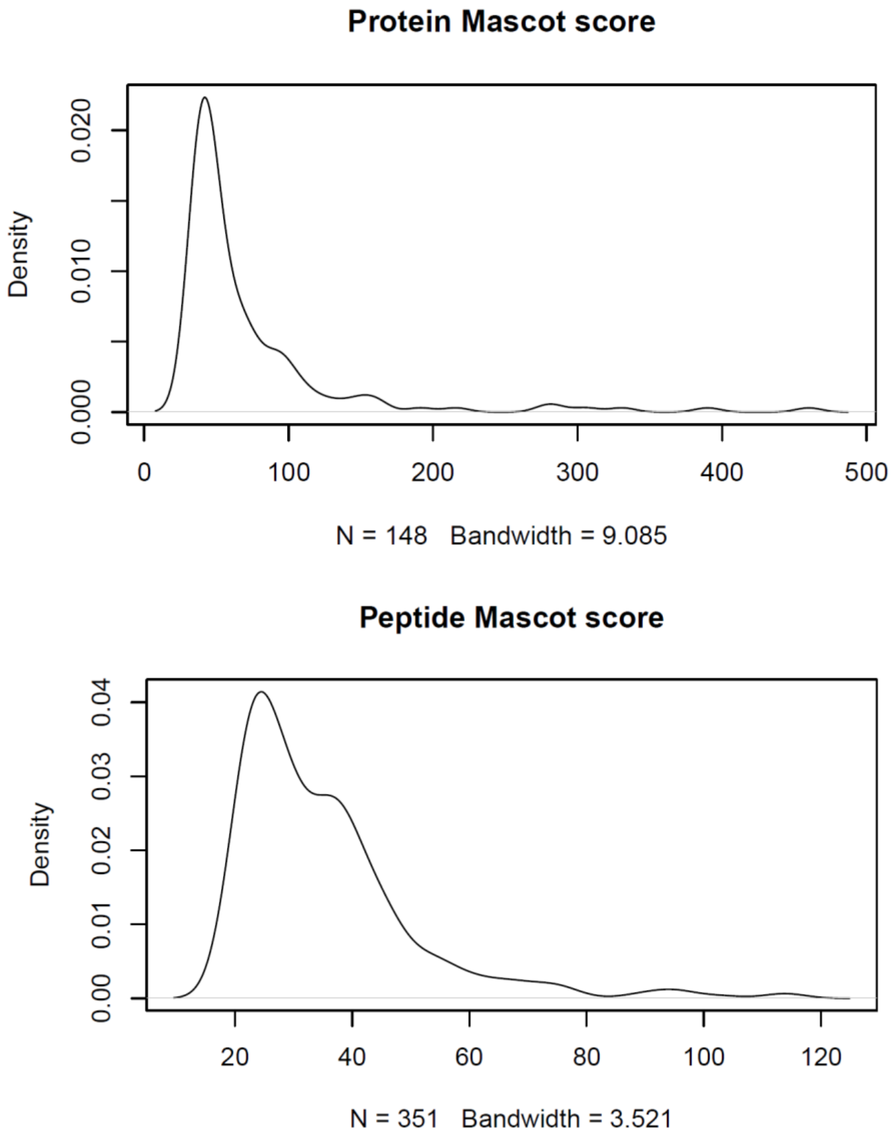

Supplementary Figure 17. nORF peptide identification. Distribution of MASCOT peptide and protein scores calculated for nORF peptides in neuropsychiatric samples using proteogenomic analysis are shown. For nORF peptide filtration, a protein score of greater than 50 and a protein expectation score of less than 0.05 was used. Following this, a peptide score of greater than 50 and peptide expectation value of less than 0.05 was used for further filtration, Finally, nORF peptides expressed in at least 30% of each case/control group were retained.

Supplementary Figure 18

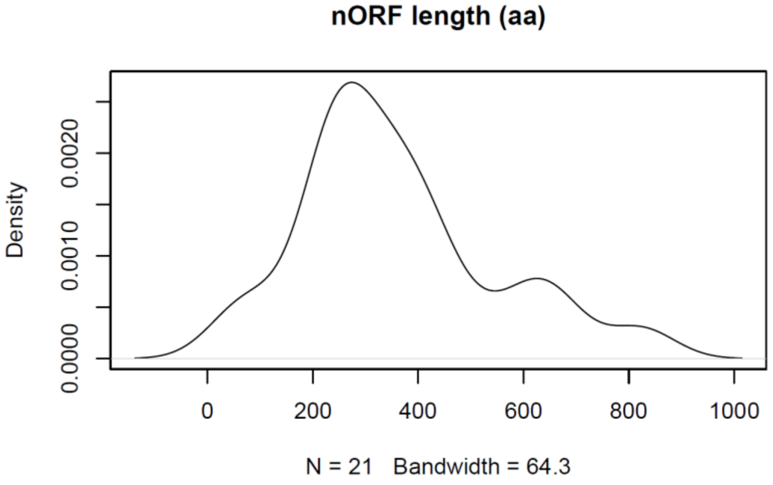

Supplementary Figure 18. nORF amino acid length distribution. For the 21 nORFs identified as translated in neuropsychiatric samples, the distribution plot above shows the length (no. of amino acids) of these nORFs. Majority of the nORFs identified are 200-300 aa in length.

Supplementary Figure 19

Supplementary Figure 19A

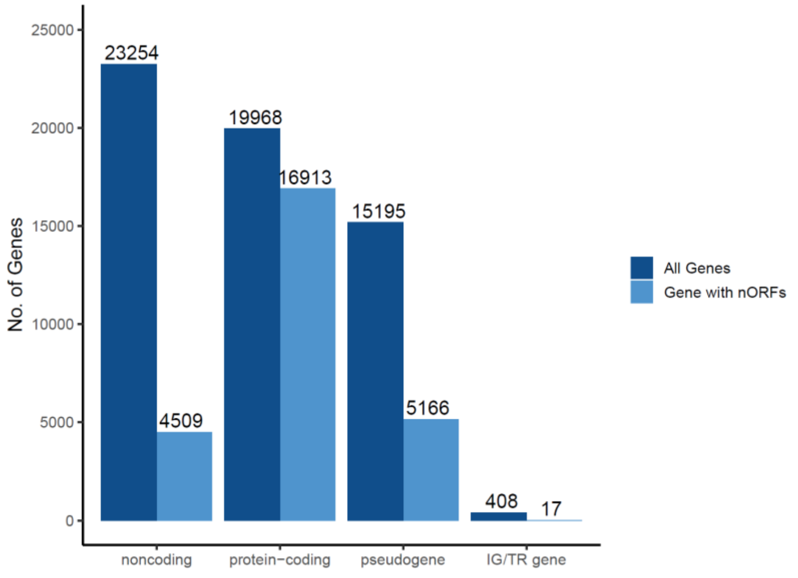

Supplementary Figure 19B

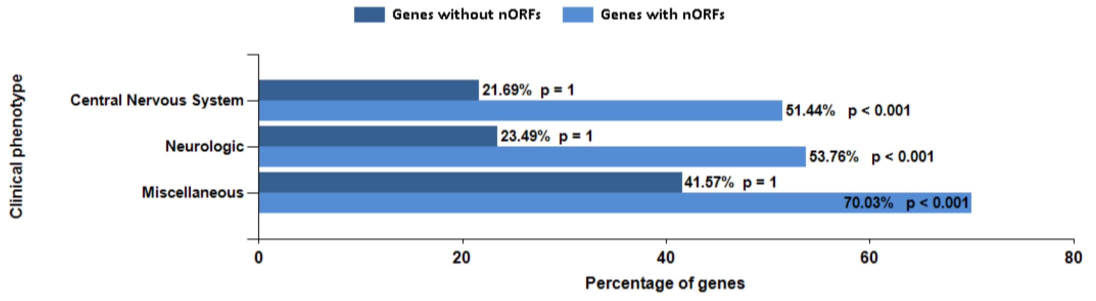

Supplementary Figure 19. Evaluation of known protein-coding genes containing nORFs. A. Number of genes with and without nORFs, split according to gene biotype is presented. nORFs are largely localized within protein-coding genes. **B.** Further evaluation of protein-coding genes was performed using FunRich v3.1.3. Percentage of genes with and without nORFs were evaluated for enrichment of clinical phenotypes. Importantly, genes with nORFs are associated significantly with neurological disorders.

Supplementary Figure 20

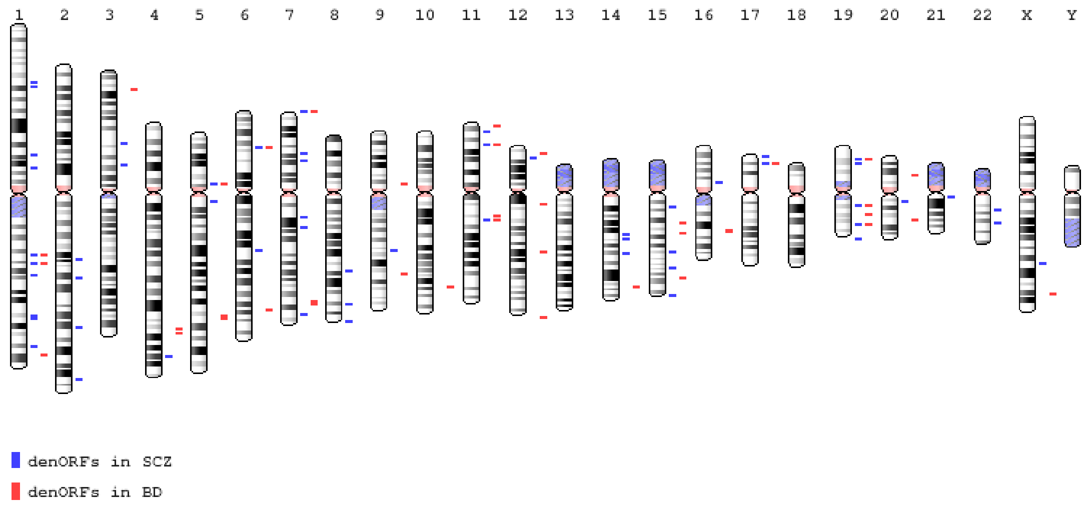

Supplementary Figure 20. An ideogram (constructed using https://www.ncbi.nlm.nih.gov/genome/tools/gdp) illustrating the location of DE nORFs within the hg19 genome assembly.

Supplementary Figure 21

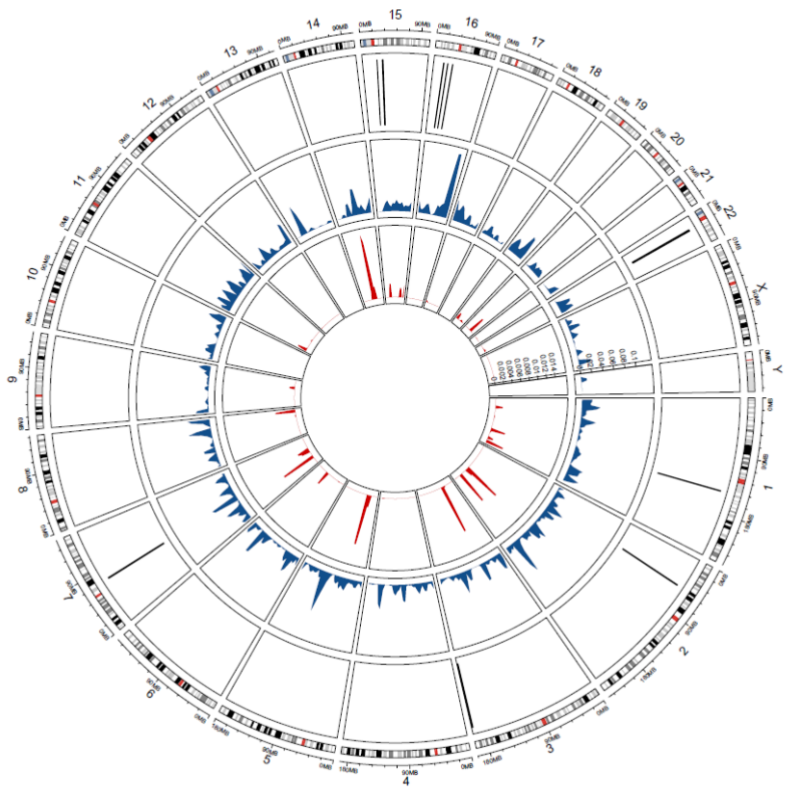

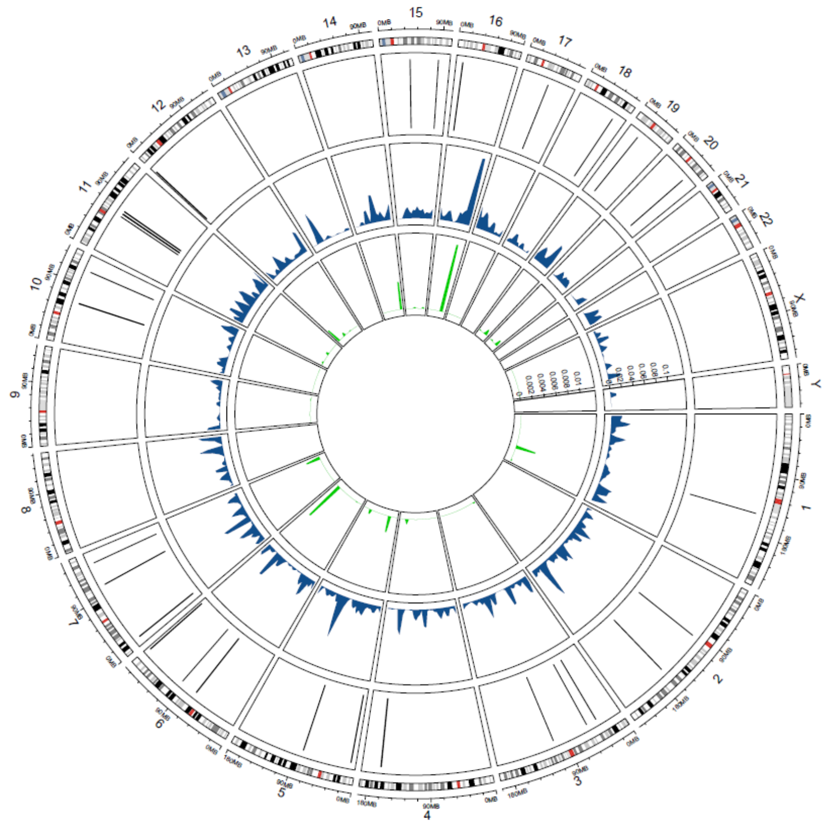

Supplementary Figure 21. nORF enrichment in BD- and SCZ- specific loci. nORFs transcribed within neuropsychiatric samples (blue peaks) and DE in SCZ (red peaks) or DE in BD (green peaks), were evaluated for overlap and enrichment within SCZ- specific CNVs and BD-specific loci (black vertical lines – outermost circular panel), using GLANET. If nORF enrichment was identified, the corresponding loci (vertical line) is marked with circular dots. For BD-specific loci and SCZ-specific CNVs (copy-number variations) showed no nORF enrichment. Enrichment was found within SCZ-specific loci (**Figure 2A**)

Supplementary Figure 22

	GWAS P-value upper bound	nORF-HARs		nORF-vHARs		nORF-mHARs		nORF-pHARs	
		P-value	Corrected P-value	P-value	Corrected P-value	P-value	Corrected P-value	P-value	Corrected P-value
Schizophrenia	e-2	9		6		3		5	
	e-3	9		6		3		5	
	e-4	9		6		3		5	
	e-5	6		4		3		4	
	e-6	0		0		0		3	
	e-7	0		0		0		2	
Bipolar disorder	e-2	1		1		1		1	
	e-3	1		1		1		1	
	e-4	1		1		1		1	
	e-5	0		0		0		0	
	e-6	0		0		0		0	
	e-7	0		0		0		0	

Legend: P < 0.001 | 0.001 < P < 0.01 | 0.01 < P < 0.05 | P > 0.05

Supplementary Figure 22: nORFs transcribed within neuropsychiatric samples were defined to be associated with a unique HAR if the unique HAR overlapped the nORF or regions extending 1 kb upstream or downstream of the nORF. All other details are as described in Figure 2C.

Supplementary Figure 23

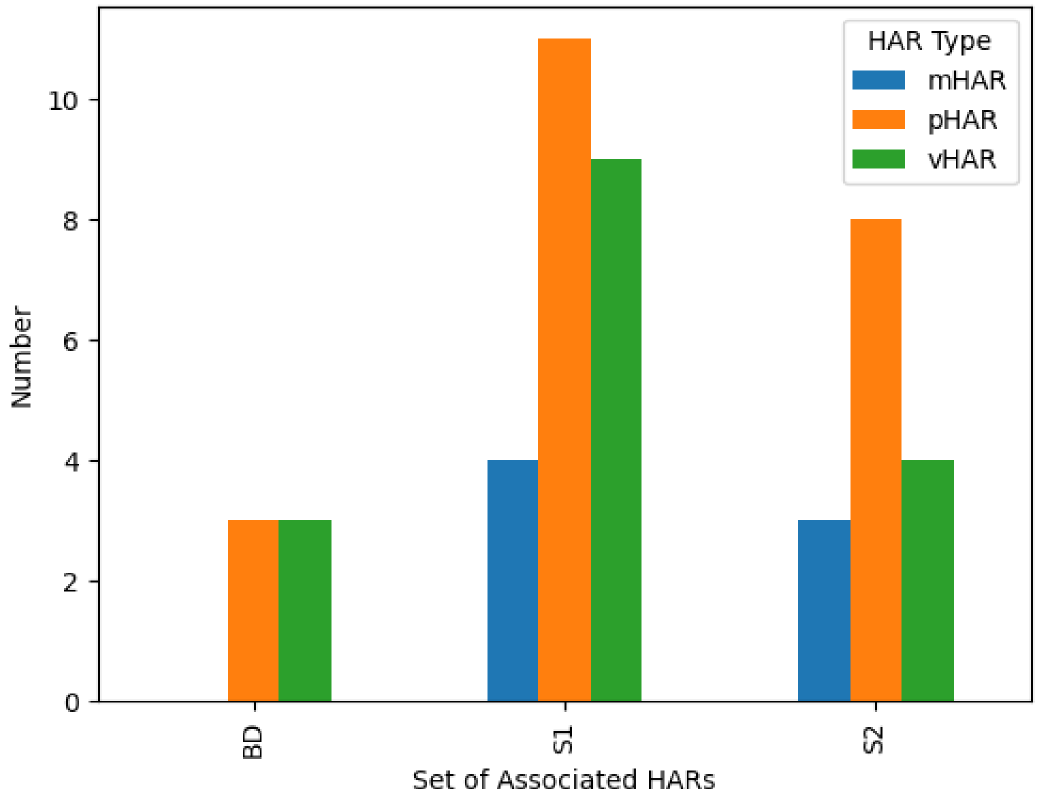

Supplementary Figure 23. Types of individual HARs within the sets of associated HARs. BD: DE nORF-HARs in BD; S1: DE nORF-HARs in SCZ; S2: DE nORF - DE HARs in SCZ.

Supplementary Figure 24

nORFID	Disorder	Classification	Chr	Nearby gene
tracer_109118	BD	nmd	9	STXBP1, syntaxin binding protein
tracer_31861	BD	utr5-cds	14	HSPA2: Heat shock protein family A (Hsp70) member 2
tracer_21834	BD, SCZ	nmd(truncation)	12	ENO2: enolase 2
dvgsH1	BD, SCZ	pseudogene	11	TUBAP2: tubulin alpha pseudogene 2 and larger DISC1FP1: DISC 1 Fusion partner 1

Supplementary Figure 24. Characterization of disorder-specific translated nORFs. Of the 21 nORFs that were identified to be translated in neuropsychiatric samples, two were uniquely expressed in BD and two in BD and SCZ. The table above highlights key features of these four nORFs including the chromosome there are present in, the nearby genes and the classification of the nORFs against the sample transcript they arise from.

Supplementary Figure 25

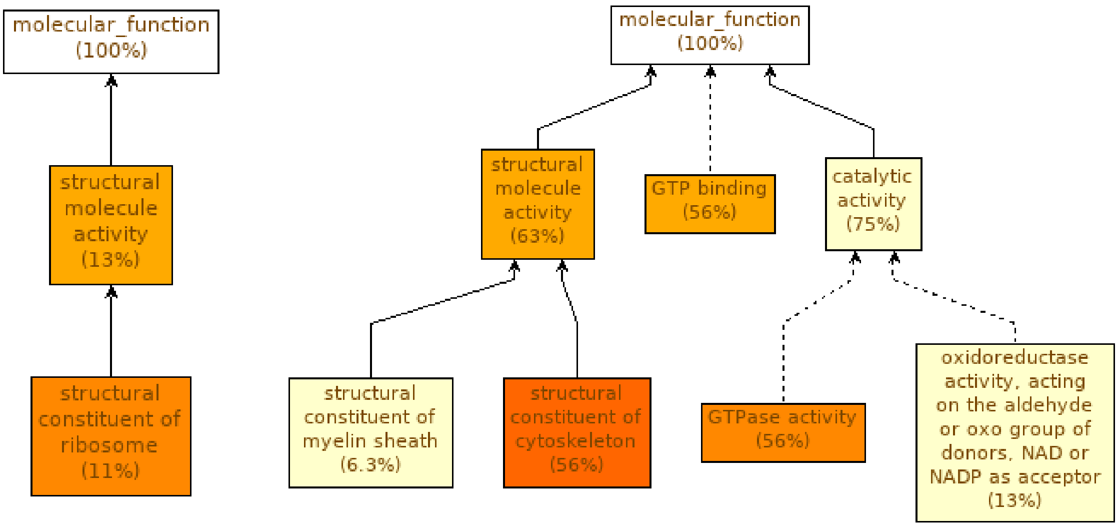

Supplementary Figure 25. GO enrichment results for transcribed and translated nORFs. For all the 248,135 nORFs used in this study, GO terms were obtained using InterProScan. For nORFs with evidence of transcription, structural molecular activity within ribosomes and therefore, potential involvement in translation was found. For nORFs that were DE, no enrichment was found possibly owing to the small set of DE nORFs with GO terms (2 in BD and 13 in SCZ). Structural molecular activity as part of the myelin sheath and cytoskeleton, GTP binding, GTPase and oxidoreductase activity were found enriched within the translated nORF set.

Supplementary Figure 26

nORF category	DHS1	Histone modification	TF
All transcribed (3103)	82	140	322
DE BD (44)	3	94	57
DE SCZ (61)	63	106	73

Supplementary Figure 26. Enrichment of genomic elements within nORFs. DHS1, histone modifications and TF enrichment within nORFs transcribed in neuropsychiatric samples (3103) and DE nORFs as evaluated using the GLANET tool.

Supplementary Figure 27

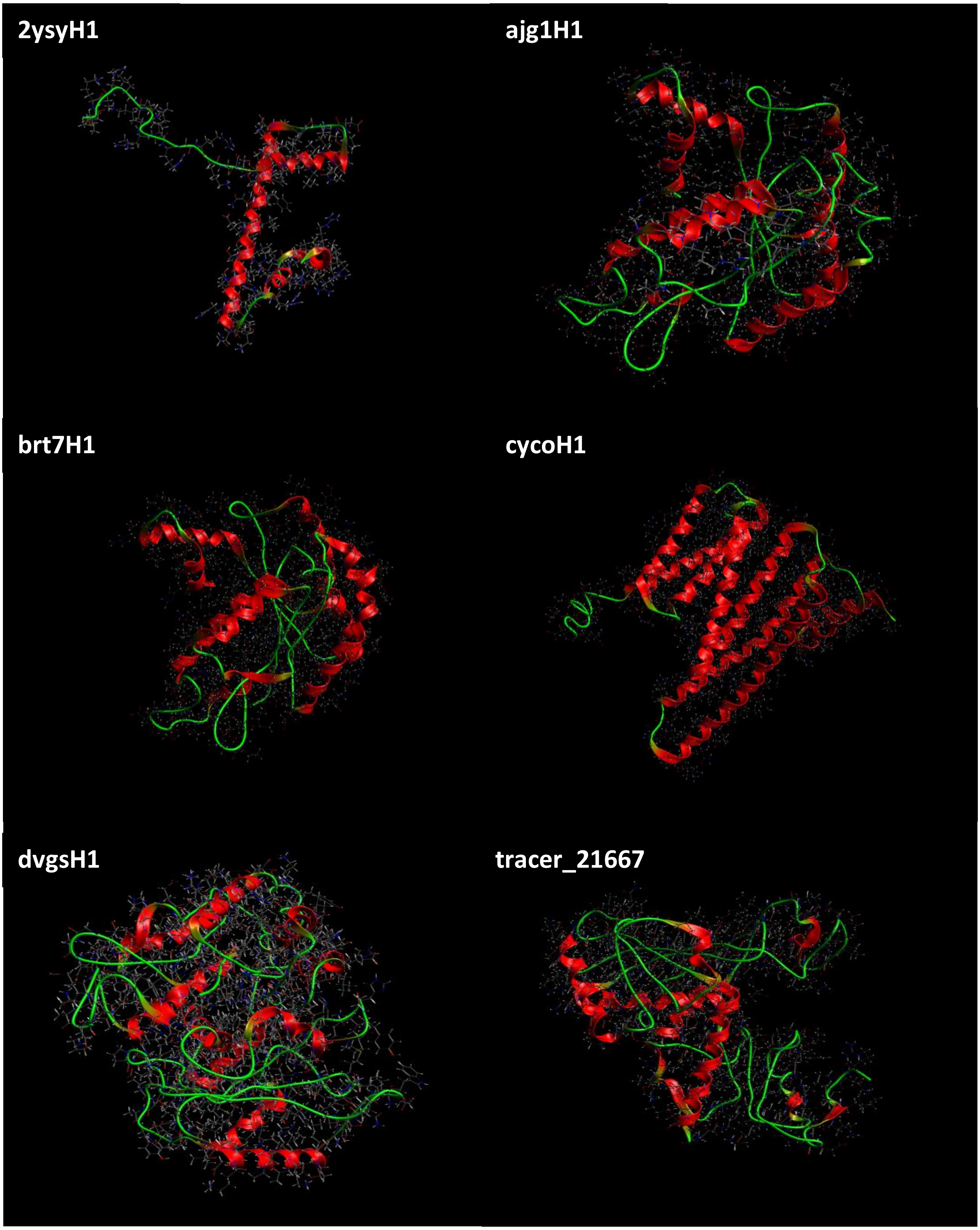

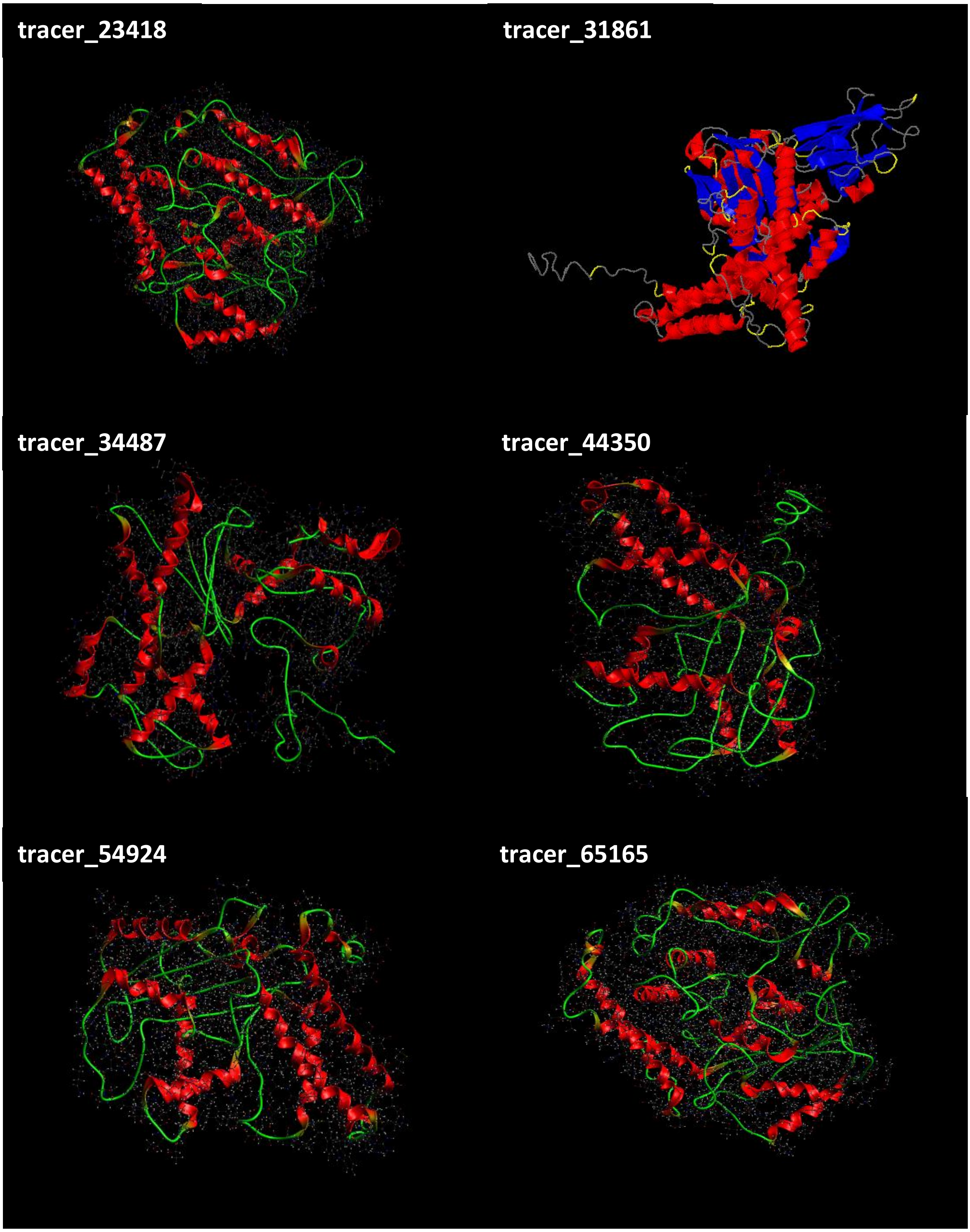
tracer_23418
tracer_31861
tracer_34487
tracer_44350
tracer_54924
tracer_65165

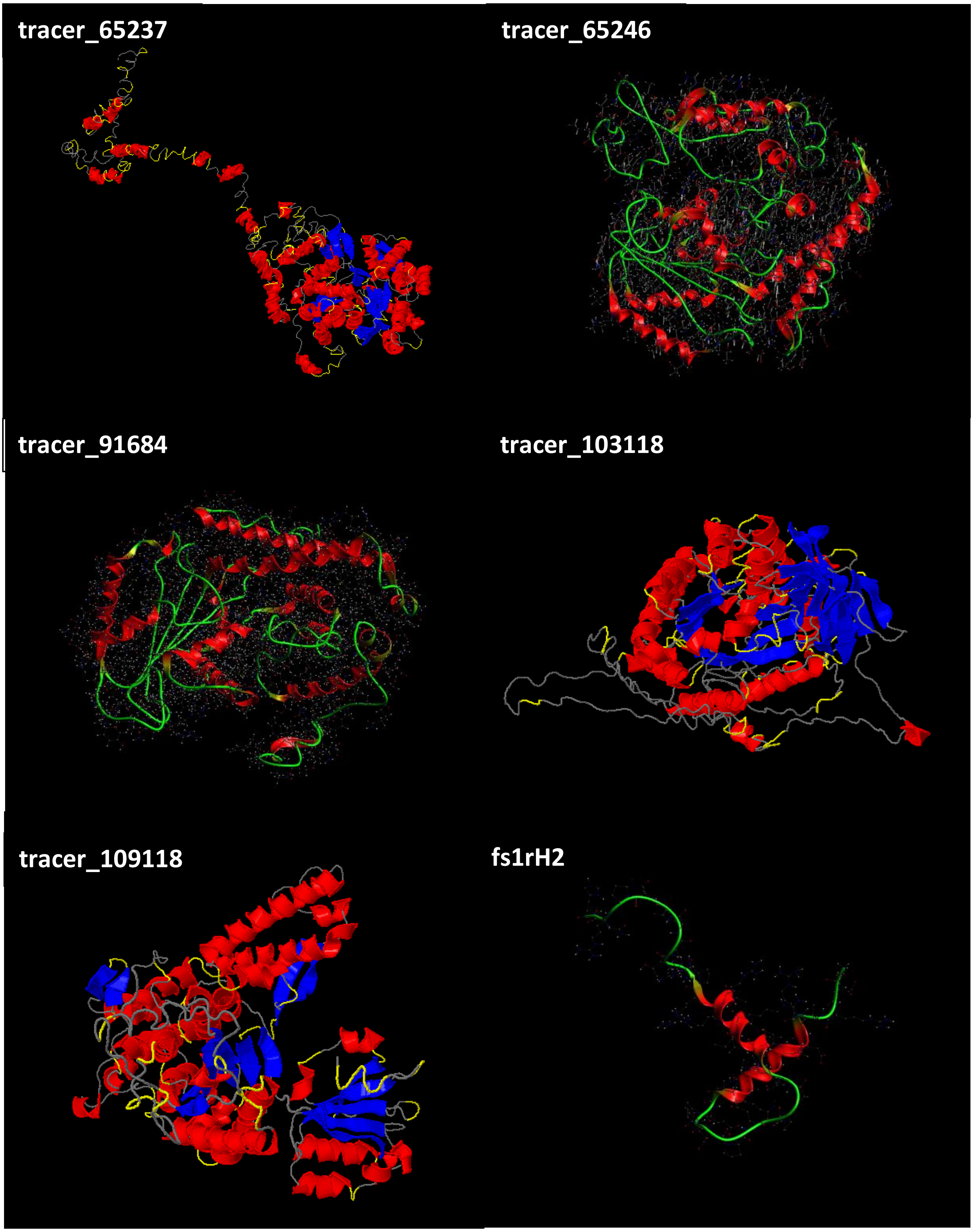
tracer_65237
tracer_65246
tracer_91684
tracer_103118
tracer_109118
fs1rH2

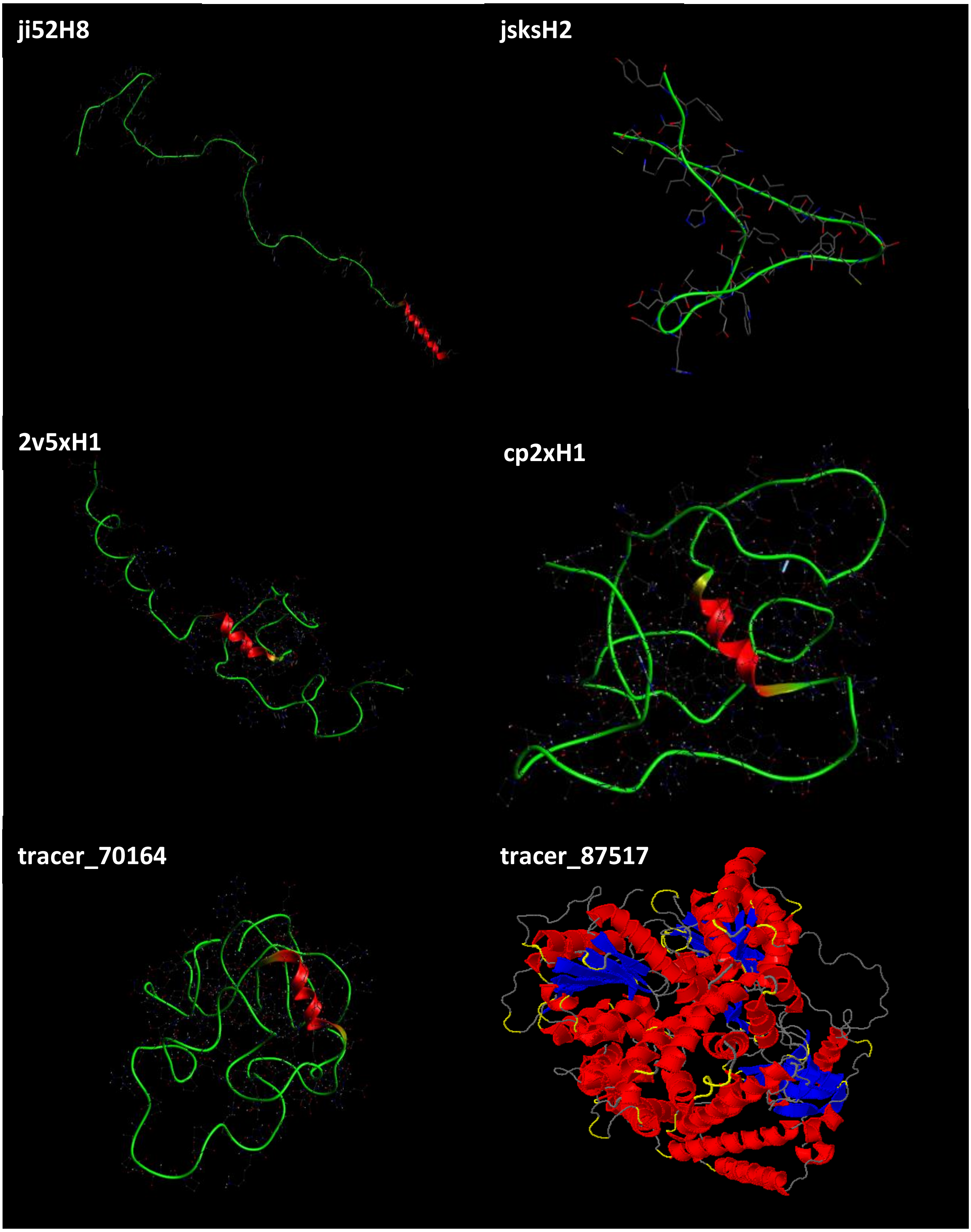
ji52H8
jsksH2
2v5xH1
cp2xH1
tracer_70164
tracer_87517

Supplementary Figure 27: Predicted structure for nORFs. Structures predicted for nORFs using i-TASSER or Raptor-X and visualised with Avogadro or Jena3D viewer. These nORFs are either translated in neuropsychiatric analysis or are associated with HARs in SCD and BD samples. To complement Figure 5. Structures for two nORFs - eveeH1 & 7t8hH4 are not shown because their corresponding amino acid length (20 and 15, respectively) is below the minimum sequence threshold required for structure prediction.
